# The relative effects of forest amount, forest configuration, and urban matrix quality on forest breeding birds

**DOI:** 10.1038/s41598-018-35276-9

**Published:** 2018-11-20

**Authors:** Alexandra Shoffner, Andrew M. Wilson, Wenwu Tang, Sara A. Gagné

**Affiliations:** 1 Department of Fisheries and Wildlife, Michigan State University, East Lansing, Michigan USA; 2 Department of Environmental Studies, Gettysburg College, Gettysburg, Pennsylvania USA; 3 Department of Geography and Earth Sciences, University of North Carolina at Charlotte, Charlotte, North Carolina USA; 4 Center for Applied Geographic Information Science, University of North Carolina at Charlotte, Charlotte, North Carolina USA

## Abstract

Urbanization modifies landscape structure in three major ways that impact avian diversity in remnant habitat: habitat amount is reduced and habitat configuration and matrix quality are altered. The relative effects of these three components of landscape structure are relatively well-studied in agricultural landscapes, but little is known about the relative effect of urban matrix quality. We addressed this gap by investigating the relative effects of forest amount, forest configuration, and matrix quality, indicated by degree of urbanization and agriculture amount, on the diversity of three guilds of forest birds using data from 13,763 point counts from Pennsylvania, USA. Forest amount had the largest independent effect on forest bird diversity, followed by matrix quality, then forest configuration. In particular, urbanization had strong negative effects on the relative abundance and species evenness of all forest birds and the relative abundance of forest generalist birds. To our knowledge, these are the first results of the effect of urban matrix quality on forest bird relative abundance and species evenness independent of forest amount and forest configuration. Our results imply that conservation practitioners in human-modified landscapes prioritize maximizing forest amount, then reducing the effects of disturbances originating in the matrix, and then preserving large, spatially-dispersed forest patches to most effectively conserve forest birds.

## Introduction

The projected doubling of developed land in the USA in the first quarter of this century^1^ was and will continue to be a significant contributor to biodiversity loss^2^. The urbanization of landscapes creates a pattern of remnant habitat patches surrounded by a matrix of residential, commercial, and industrial land uses. Three major aspects of landscape structure have important effects on avian diversity in remnant habitat in urban landscapes: habitat amount, habitat configuration, and matrix quality.

Habitat amount is the total area of remnant habitat in a landscape. Declines in biodiversity due to decreasing habitat amount are well-documented across taxa and regions^3^. For example, Smith *et al*.^4^ found that total habitat amount had strong and consistently positive effects on the presence of forest birds within human-altered landscapes. Habitat amount has been hypothesized to positively influence biodiversity by means of passive sampling, higher habitat diversity, and lower extinction rates due to larger population sizes or less frequent and intense disturbances^5^.

Habitat configuration is the spatial arrangement of habitat in a landscape, independent of habitat amount (Fahrig’s^6^ fragmentation per se). Habitat configuration has somewhat equivocal effects on biodiversity. Empirical studies show that the independent effects of configuration are generally weak and may be positive or negative^6,7^. For example, Villard *et al*.^8^ found that the number of forest fragments had a significant positive effect on Veery (*Catharus fuscescens)* occurrence, whereas fragment mean nearest-neighbor distance had a significant negative effect on Scarlet Tanager (*Piranga olivacea*) occurrence. Habitat configuration has been hypothesized to affect biodiversity through a number of different mechanisms: the reduced persistence of small, isolated populations in small patches, negative or positive edge effects, increased predator-prey system stability through the provision of prey refugia in small patches, the enhanced co-existence of competing species, the reduced probability of simultaneous extinction of all subpopulations, higher immigration rate as a function of increased edge, increased colonization success due to smaller distances among patches, and landscape complementation^6^.

Matrix quality is the degree to which human activities in the matrix disturb natural processes^9^. As such, matrix quality is a major determinant of biodiversity in human-dominated landscapes^10^. For example, the abundance of native forest birds in forest patches is lower within intensely urban landscapes (low matrix quality) than within suburban or exurban landscapes (higher matrix quality)^11^. Three mechanisms have been hypothesized to explain the effect of matrix quality on biodiversity^12^. First, matrix quality may be indicative of dispersal mortality, e.g., collisions with buildings are a major source of mortality for birds^13^. Second, matrix quality may encompass variation in the availability of resources that are supplemental to those in habitat patches, such as bird feeders^14^. Finally, variation in matrix quality may be associated with varying disturbance occurrence, intensity, and frequency in habitat patches, such as human trail use^15^.

The relative effects of these three aspects of landscape structure on biodiversity are of great interest because they can directly inform the effectiveness of management and planning for species conservation in human-altered landscapes^9^. Though there is broad support for habitat amount being a more important determinant of biodiversity than habitat configuration^4,6,16–23^, there is less consensus regarding the relative effect of matrix quality. Existing evidence points to matrix quality having an effect on biodiversity smaller than that of habitat amount but larger than that of habitat configuration^18,24–28^.

Although some of the empirical studies that have compared the relative effects of these aspects of landscape structure have been carried out in landscapes altered by urban development (e.g.^23^), only one has included an explicitly urban indicator of matrix quality in analyses, i.e., amount of developed land^29^. Matrix quality in other studies has been assessed in terms of the influence of agricultural land uses and/or roads^4,18,19,22,23,25^ or manipulated in small-field experiments^17,30,31^. Therefore, there is a significant need for research investigating the relative impacts of habitat amount, habitat configuration, and urban matrix quality.

In this article, we asked the following research question: What are the relative effects of habitat amount, habitat configuration, and matrix quality, including urban matrix quality, on the diversity of forest birds? Based on the strength of evidence to date, we predicted that habitat amount would have the largest effect, followed by matrix quality and habitat configuration, in that order. We addressed our research question by quantifying forest amount, forest configuration, and two measures of matrix quality, degree of urbanization and agriculture amount, at multiple scales in 13,763 landscapes across the state of Pennsylvania, USA. For each landscape, we estimated the relative abundance, species richness, and species evenness of three habitat guilds of forest birds – all forest birds, forest-area sensitive birds, and forest generalist birds – using data from the Second Pennsylvania Breeding Bird Atlas^32^. We determined the independent effects and relative importance of each of our three components of landscape structure of interest, controlling for the confounding effects of local habitat quality, landscape heterogeneity, and species detectability, using general linear modeling.

## Results

Landscape variables in the best models of forest bird diversity were measured at four spatial scales: 0.2 km for models of all forest bird relative abundance, species richness, and species evenness and forest generalist bird species evenness; 1 km for the best model of forest-area sensitive bird species richness; 6 km for models of the relative abundance and species evenness of forest-area sensitive birds and the relative abundance of forest generalist birds; and, 10 km for the best model of forest generalist bird species richness (see Supplementary Tables S1–S9).

**Figure 1:**
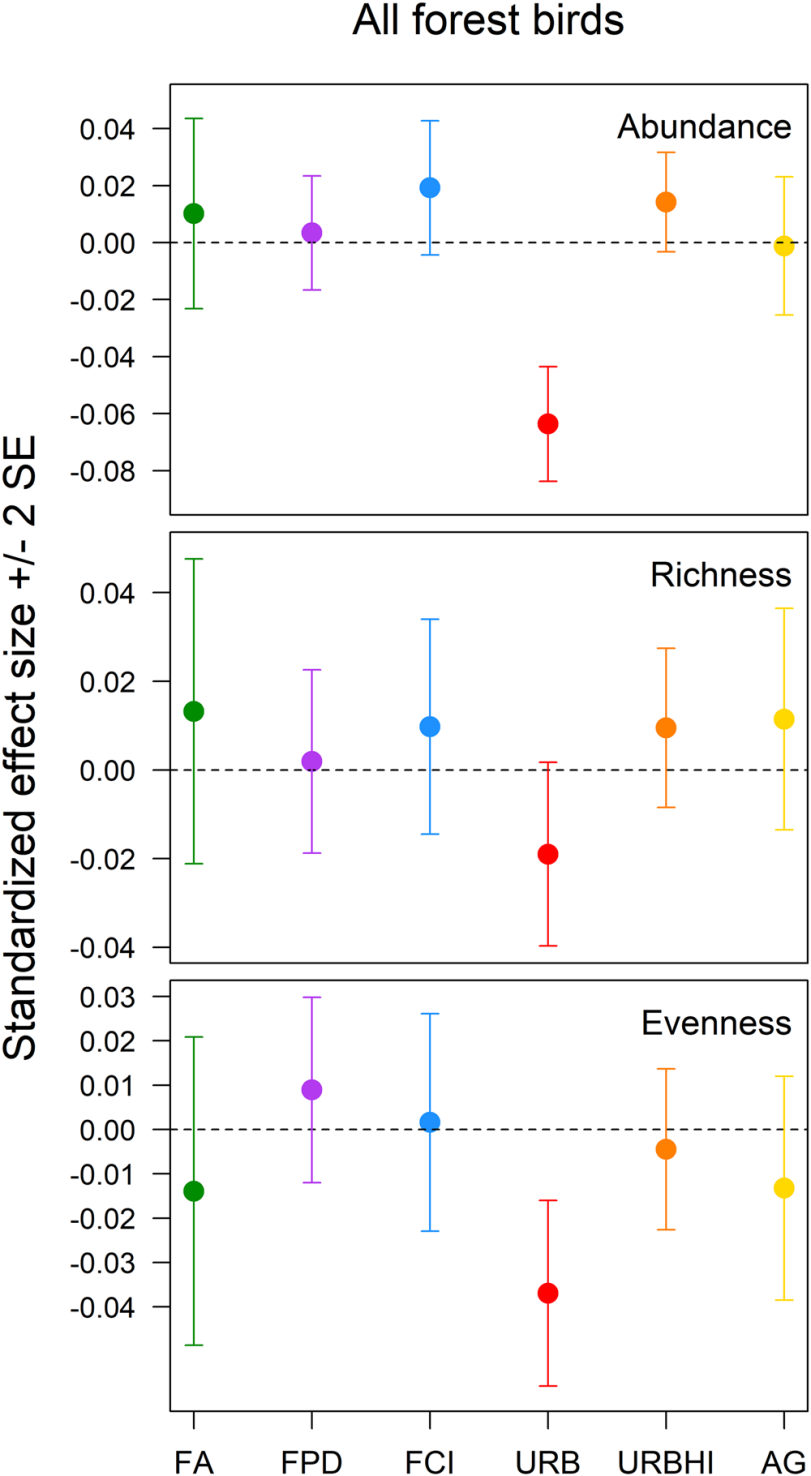
The standardized effects (±2 SE) of forest amount (FA), forest patch density (FPD), forest clumpiness index (FCI), urbanization (URB), high intensity urbanization (URBHI), and agriculture amount (AG) on the relative abundance, species richness, and species evenness of the all forest breeding bird guild in Pennsylvania, USA.

**Figure 2:**
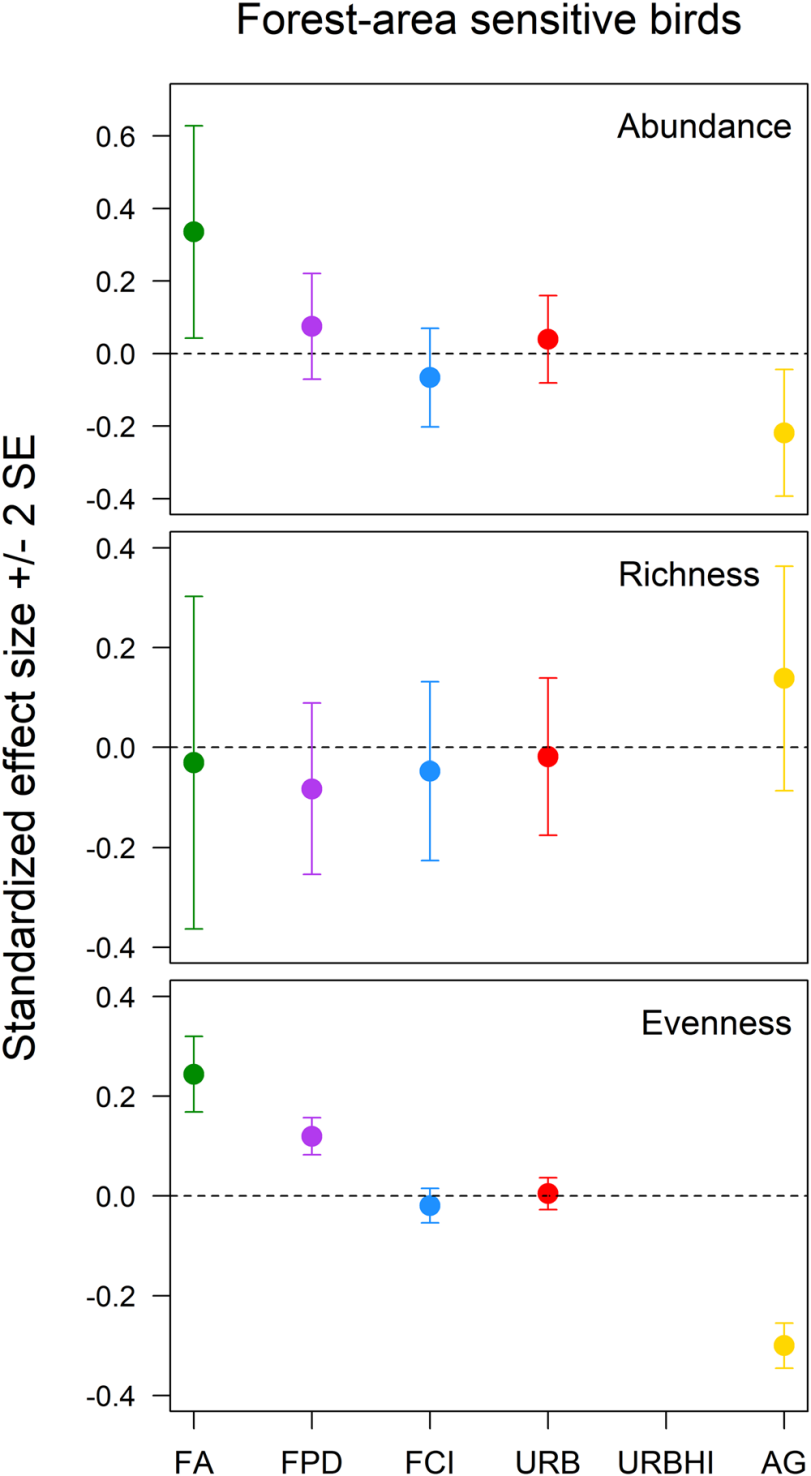
The standardized effects (±2 SE) of forest amount (FA), forest patch density (FPD), forest clumpiness index (FCI), urbanization (URB), high intensity urbanization (URBHI), and agriculture amount (AG) on the relative abundance, species richness, and species evenness of the forest-area sensitive breeding bird guild in Pennsylvania, USA.

**Figure 3:**
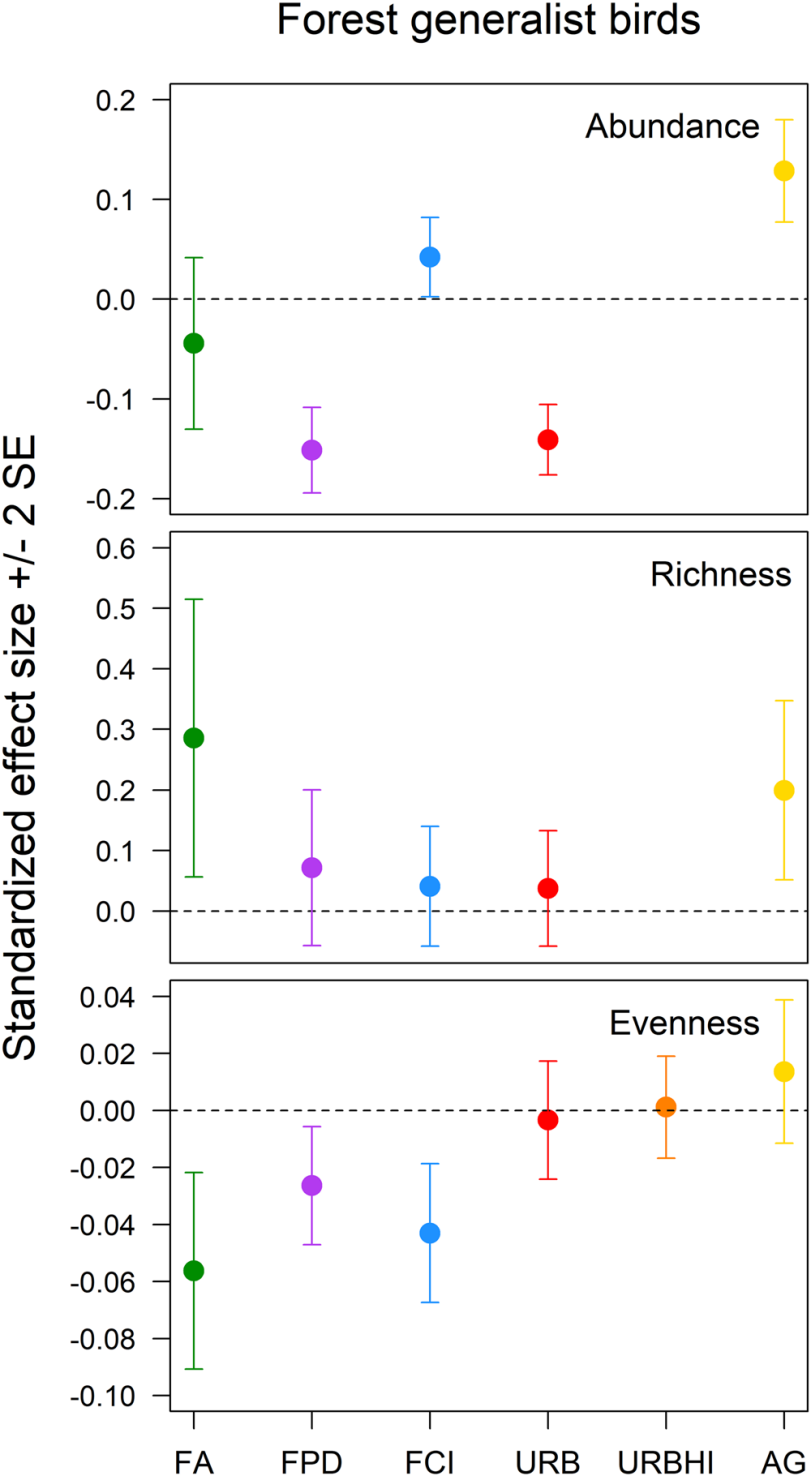
The standardized effects (±2 SE) of forest amount (FA), forest patch density (FPD), forest clumpiness index (FCI), urbanization (URB), high intensity urbanization (URBHI), and agriculture amount (AG) on the relative abundance, species richness, and species evenness of the forest generalist breeding bird guild in Pennsylvania, USA.

Urbanization was the only landscape structure variable of interest that had a meaningful effect on the diversity of all forest birds (relative abundance: β (95% CI) = −0.06 (−0.08, −0.04); species evenness: β (95% CI) = −0.04 (−0.06, −0.02)) (Fig. 1, see Supplementary Figs S1, S2). The diversity of forest-area sensitive birds was positively affected by forest amount (relative abundance: β (95% CI) = 0.34 (0.04, 0.63); species evenness: β (95% CI) = 0.24 (0.17, 0.32)) and forest patch density (species evenness: β (95% CI) = 0.12 (0.08, 0.16)) and negatively affected by agriculture amount (relative abundance: β (95% CI) = −0.22 (−0.39, −0.04); species evenness: β (95% CI) = −0.30 (−0.35, −0.25)) (Fig. 2, see Supplementary Figs S3–S7). The relative abundance of forest generalist birds was positively influenced by agriculture amount (β (95% CI) = 0.13 (0.08, 0.18)) and forest clumpiness index (β (95% CI) = 0.04 (0.002, 0.08)) and negatively influenced by forest patch density (β (95% CI) = −0.15 (−0.19, −0.11)) and urbanization (β (95% CI) = −0.14 (−0.18, −0.11)) (Fig. 3, see Supplementary Figs S8–S11). Forest generalist species richness was positively influenced by forest amount (β (95% CI) = 0.29 (0.06, 0.52)) and agriculture amount (β (95% CI) = 0.20 (0.05, 0.35)) (Fig. 3, see Supplementary Figs S12, S13). Finally, the species evenness of forest generalist birds was negatively affected by forest amount (β (95% CI) = −0.06 (−0.09, −0.02)), forest patch density (β (95% CI) = −0.03 (−0.05, −0.01)), and forest clumpiness index (β (95% CI) = −0.04 (−0.07, −0.02)) (Fig. 3, see Supplementary Figs S14–S16).

Considering the effects just described, forest amount ranked first in importance to forest bird diversity relative to other components of landscape structure, having the absolute largest meaningful effects on three diversity measures (forest-area sensitive bird relative abundance and forest generalist bird species richness and species evenness) and the absolute second largest meaningful effect on an additional measure (the species evenness of forest-area sensitive birds) (Figs 2 and 3). The meaningful effects of matrix quality variables (agriculture amount and urbanization) on forest bird diversity were just as often the absolute largest in magnitude (the relative abundance and species evenness of all forest birds and forest-area sensitive bird species evenness) as the absolute second largest (the relative abundances of forest-area sensitive birds and forest generalist birds and the species richness of forest generalist birds) (Figs 1–3). Forest configuration variables (forest patch density and forest clumpiness index) typically ranked third in importance relative to forest amount and matrix quality variables, having either the absolute smallest meaningful effect or meaningless effects on forest bird diversity (forest-area sensitive bird relative abundance and species evenness and forest generalist bird species richness) (Figs 2 and 3).

## Discussion

Forest amount was the most important determinant of patterns of forest bird diversity in our data, followed by matrix quality and forest configuration. This order of importance matches our prediction based on the findings of a large majority of studies on the species^4,6^ and (references therein^16,18,20^). Forest amount typically had strong positive effects on the diversities of forest-area sensitive and forest generalist birds, which may be explained by passive sampling or lower extinction rates. Matrix quality variables also exhibited strong effects, particularly the negative effects of urbanization on the diversity of all forest birds and the negative effect of agriculture amount on forest-area sensitive bird species evenness. These effects are likely a result of disturbances associated with urbanization and agricultural practices that reduce habitat quality for forest birds and increase dispersal mortality. In comparison, forest configuration was the least important component of landscape structure, particularly to forest-area sensitive birds. These species preferentially occur in landscapes with large amounts of forest where variation in the configuration of forest may be restricted.

According to our results, landscapes with more forest cover in Pennsylvania should host more forest generalist bird species and a greater relative abundance and species evenness of forest-area sensitive birds. The Passive Sampling Hypothesis, the Theory of Island Biogeography, and the Disturbance Hypothesis predict just such outcomes^5^. The Passive Sampling Hypothesis assumes that the individuals in a community are randomly distributed within habitat in a region. A larger sample or area of habitat should contain a larger sample of the community’s individuals and species. The Theory of Island Biogeography predicts an increase in species number with island size as a function of the lower extinction rates of larger populations on larger islands. Larger populations of a variety of species may explain the positive effect of forest amount on species evenness in our data. The Disturbance Hypothesis posits that the frequency and intensity of disturbances diminish on larger islands, thereby leading to lower extinction rates. A fourth explanation for the positive effect of habitat amount on species number is the Habitat Diversity Hypothesis that states that niche diversity increases with habitat area. We discount this hypothesis in the present context because we controlled for habitat diversity in our models by including measures of landscape heterogeneity that accounted for the diversity of land cover types, including forest types, in landscapes. Our results also included a negative effect of forest amount on the species evenness of forest generalist birds. Some forest generalist bird species, such as the Prairie Warbler (*Setophaga discolor*) and the Louisiana Waterthrush (*Parkesia motacilla*), may make use of habitat types in addition to forest, such as shrubland and wetland. For a given area of agriculture and degree of urbanization in a landscape, an increase in forest cover results in a decrease in the covers of other habitat types, potentially negatively impacting a subset of forest generalist bird species and leading to lower species evenness for the group.

Urbanization had negative effects on the relative abundance and species evenness of all forest birds and the relative abundance of forest generalist birds. We attribute these results to the cumulative effects of the variety of disturbances associated with urbanization that act to increase avian mortality and decrease reproductive productivity. Urbanization is accompanied by increases in buildings, roads, and power lines^33^ and domestic cat (*Felis catus*) densities^34^, the four most common sources of mortality for birds in North America^13^. Urban development is also associated with increased air pollution, higher air temperatures, human intrusion into remnant habitat, introduced plant species occurrence, higher brood parasite density, greater noise, and lower caterpillar abundance^15,35–39^. These factors have been shown to negatively impact the presence of breeding territories, hatching success, chick survival, the number of fledglings per nesting attempt, and chick and fledgling weight, and positively affect nest predation rate^35,40–44^.

Agriculture amount was just as likely to have negative as positive effects on forest bird diversity. Forest-area sensitive bird relative abundance and species richness declined with increasing agriculture amount in landscapes, whereas forest generalist bird relative abundance and species richness increased. Forest-area sensitive bird species were defined as requiring large amounts of forest in landscapes that, because of the positive association between forest amount and forest aggregation in our data, were likely more continuous. Thus, forest-area sensitive birds may also be considered as species adapted to interior forest conditions. As such, they would be particularly vulnerable to the negative edge effects associated with agriculture due to elevated nest parasitism and predation rates^45^ and exposure to herbicides and pesticides^46–48^. Forest-area sensitive birds may also be particularly vulnerable to dispersal mortality in an agricultural matrix. Species that evolved in landscapes with continuous habitat and little or no matrix exhibit weak boundary responses and tortuous movement, characteristics that make them more prone to dispersal mortality in the matrix following habitat loss compared to species that evolved in patchy habitat^49^. Forest generalist birds, on the other hand, clearly benefited from the additional resources provided by agriculture. For example, approximately a quarter of our forest generalist bird species are predators of insect pests in crop fields and pastures, e.g., Northern Parula (*S. americana*)^50^.

Habitat configuration was the least important component of landscape structure affecting forest bird diversity in our study. This result adds to the existing evidence that the configuration of habitat, independent of habitat amount or matrix quality, exerts the smallest relative influence on forest bird diversity^16,18,20^ and the diversity of other taxa^6^ and (references therein^17,21,24,25,31^). The lesser importance of habitat configuration with respect to habitat amount has been explained by the Habitat Amount Hypothesis, which states that the major determinant of species richness at a sampling site is the amount of surrounding habitat, by means of passive sampling, and that habitat configuration is unimportant^51^. Our results support this hypothesis in that forest configuration variables were less important to forest generalist bird species richness than forest amount. Forest configuration variables were also of lesser importance than forest amount and matrix quality variables to forest-area sensitive bird relative abundance and species evenness, which may seem surprising given that this guild is associated with continuous forest (see above). We suggest that, in the landscapes in which forest-area sensitive bird species preferentially occur, i.e., those with large amounts of continuous forest, there is necessarily less variation in forest configuration, and, as a consequence, a reduced likelihood of large meaningful effects of our configuration variables. We did find, however, relatively large effects of forest configuration variables on the diversity of forest generalist birds. Forest generalist bird relative abundance was higher in landscapes with lower forest patch densities or higher clumpiness, or spatial aggregation, of forest cover. Species evenness of the guild increased with decreasing forest patch density or decreasing forest aggregation. These results suggest that forest generalist species populations are larger in larger forest patches (for a given forest amount in a landscape, lower patch density implies larger patches) and more evenly so when forest is spatially dispersed. The dispersion of forest, manifested as increasing inter-patch distances and/or increasingly irregular and elongated patch shapes, increases the interdigitation of forest and other land covers, thereby facilitating landscape complementation for the subset of forest generalist species that make use of multiple habitat types.

The relative importance of components of landscape structure on biodiversity is likely to be contingent on the diversity measure and indicators of landscape structure used in any given study. In the present study, the order of importance of forest amount, matrix quality, and forest configuration differed among diversity measures, with only two measures, forest-area sensitive bird relative abundance and forest generalist bird species richness, exhibiting the same ordering as forest bird diversity overall. The order of importance of our landscape structure components of interest would also differ if we had used a limited subset of landscape structure variables. For example, matrix quality would have been the least important determinant of overall forest bird diversity if we had not included agriculture amount in analyses, whereas it would have been the most important if we had not included forest patch density. Similarly, there may be indicators of matrix quality for forest birds that we did not measure, such as road density^4^, whose inclusion in future analyses may result in different conclusions than those presented here. Thus, it may be difficult to predict the relative importance of habitat amount, habitat configuration, and matrix quality for any given measure of biodiversity and set of landscape structure indicators.

The overall order of importance of forest amount, matrix quality, and forest configuration that we report implies that planners and managers prioritize maximizing the amount of forest, irrespective of its spatial configuration, to most effectively conserve forest birds. If this is not feasible, e.g., in multi-use landscapes with high development pressure, planners and managers should focus on minimizing the effects of disturbances originating in the matrix that negatively impact habitat quality in forest remnants and increase dispersal mortality. This could involve educational campaigns, incentives, and/or bylaws to keep cats indoors, make windows more visible to birds, and reduce pesticide and herbicide use, road traffic, impervious surface cover, introduced plant species cover, and recreation in forest remnants. Finally, if maintaining or increasing forest amount and/or limiting disturbances originating in the matrix are not possible, the focus should be on preserving large forest patches that are elongate or irregularly-shaped and/or isolated from one another. This sequential implementation of conservation measures based on the relative effects of components of landscape structure is important. It ensures that planners and managers are implementing the most effective conservation measure, relative to those evaluated, given the limits of time and funding and the demands of other, often competing goals, such as building housing.

In conclusion, we report that forest amount had the largest independent effect on forest bird diversity, followed by matrix quality and forest configuration. Forest amount generally had positive effects on forest bird diversity. Urbanization had negative effects on the relative abundance and species evenness of all forest birds and the relative abundance of forest generalist birds. To our knowledge, these are the first results of the effect of urban matrix quality on forest bird relative abundance and species evenness independent of forest amount and forest configuration. Agriculture amount had negative effects on the diversity of forest-area sensitive birds and positive effects on the diversity of forest generalist birds. Finally, forest patch density generally had negative effects on forest bird diversity, whereas forest clumpiness had positive and negative effects. The order of importance of forest amount, matrix quality, and forest configuration to forest bird diversity implies that planners and managers with the goal of conserving forest birds in landscapes modified by urbanization and agriculture prioritize maximizing forest amount, then reducing the effects of disturbances originating in the matrix, and then preserving large, spatially-dispersed forest patches.

## Methods

### Study Area

Pennsylvania has an area of 119,283 km^2^ and intersects three Bird Conservation Regions: the Appalachian Mountains, the Lower Great Lakes, and the Piedmont^52^. Mountains (maximum elevation = 979 m a.s.l.) are covered by northern hardwood forest, whereas agriculture and urban development dominate lower elevations (minimum elevation = 0 m a.s.l.). Over half of the state’s population of 12.7 million people is concentrated in the metropolitan areas of Philadelphia and Pittsburgh in the southeast and southwest corners of the state, respectively^53^. Developed land in Pennsylvania more than doubled between 1992 and 2005, primarily at the expense of open space and agricultural land surrounding urban areas^53^.

### Second Pennsylvania Breeding Bird Atlas

We used point count data from the Second Pennsylvania Breeding Bird Atlas (PBBA)^32^. Between 2004 and 2009, 22 trained PBBA staff performed 33,763 unlimited-distance roadside point counts, resulting in the observation of 176 bird species. Approximately eight point counts were conducted in each of 4,937 ‘blocks’, each approximately 24 km^2^ in area, covering the state. Points were situated at random locations that were shifted to the nearest non-highway road, while maintaining a minimum 400 m distance between points^54^. Counts occurred between May 25 and July 4 each year in suitable weather conditions between 5 and 10 am, with each point being visited once over the atlas period for 6 minutes and 15 seconds.

### Landscape Selection

**Figure 4:**
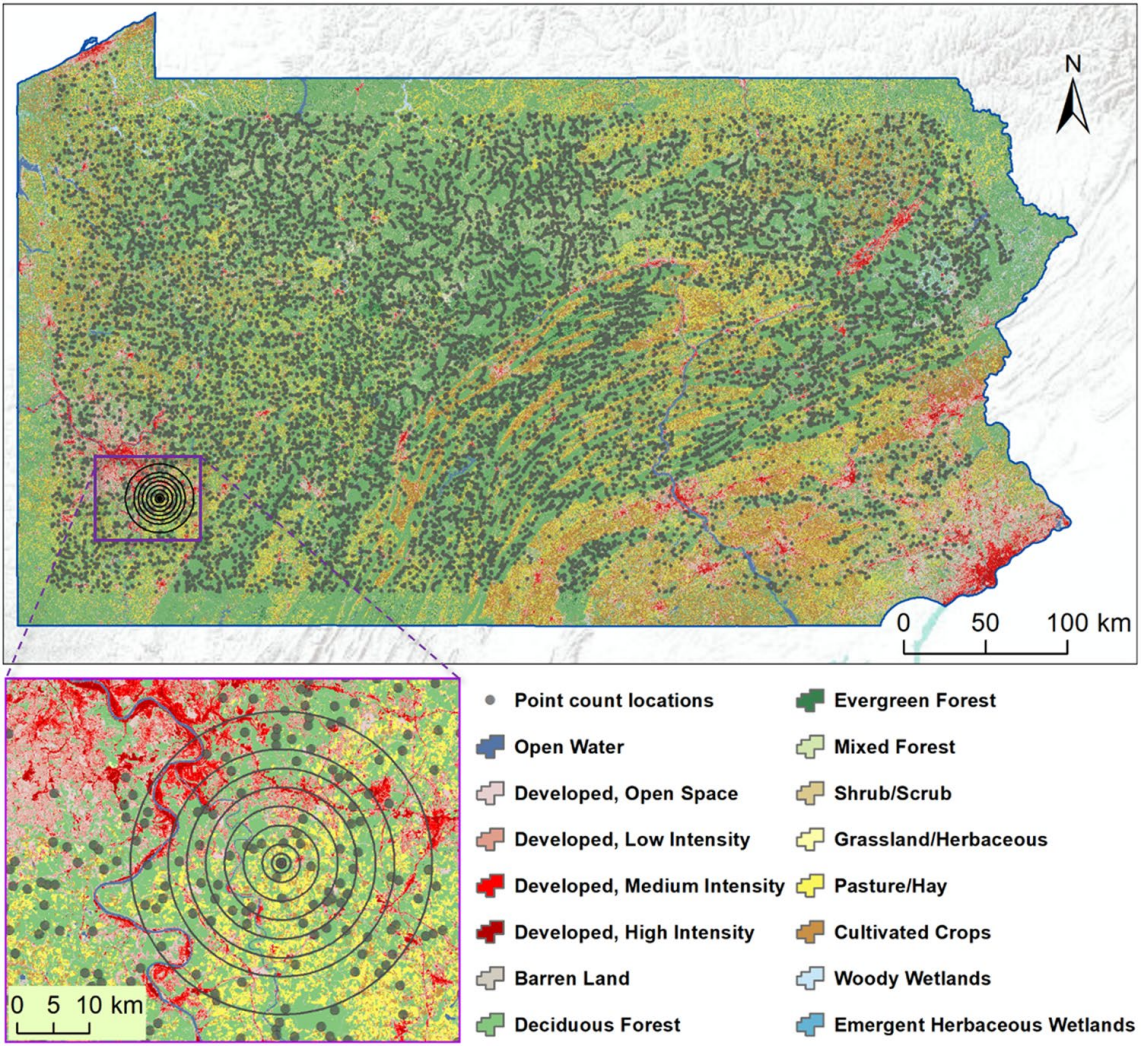
Locations of the 13,763 point counts selected from the 2^nd^ Pennsylvania Breeding Bird Atlas, USA. The inset depicts the landscapes of ten different scales surrounding each count location. Land cover is from the National Land Cover Database^74^.

Landscapes were defined as circular areas centered on point count locations that were surrounded by ≥50% forest cover within a 0.2 km radius. We chose ten landscape radii (0.2, 0.5, 1, 2, 4, 6, 8, 10, 12, and 16 km) to account for scale-dependent variability in the relative importance of habitat amount, habitat configuration, and matrix quality^4^. This range of scales generally encompassed the median natal dispersal distances and territory or home range sizes of the non-raptor and non-waterfowl species observed during the PBBA at the count locations that we selected^55–59^, as well as the scales at which habitat amount, habitat configuration, and matrix quality have been found to affect forest bird diversity^4,16,18,20^. In addition, we considered a wide range (1.9 orders of magnitude) and high density (0.6 scales/km) of scales in order to improve the likelihood that we identified the true scales of effect of our landscape variables of interest^60^. In our selection of point count locations, we omitted those that were ≤16 km from the state border to ensure that all selected points were surrounded by entire landscapes at all scales. These selection criteria resulted in 13,763 landscapes at each of 10 scales (Fig. 4).

### Forest Bird Diversity

We excluded species from the 176 detected at selected points that had the following characteristics: (1) hybrid species; (2) irregular breeder in the state; (3) raptor; (4) waterfowl; and (5) occurrence at fewer than 30 count locations. The resultant selection of 101 species was divided into three habitat association guilds using species’ empirical patterns of occurrence across landscape scales. Avian responses to forest configuration have been shown to vary both regionally^61–63^ and temporally^64^. For example, the effects of forest configuration on the Wood Thrush (*Hylocichla mustelina*)^65^ and Scarlet Tanager have been shown to vary regionally^66,67^, which might explain why evidence for area-sensitivity can be equivocal for some species (e.g.,^68^). Our empirical approach avoided such ambiguities by ensuring that guild definitions were accurate in the context of our study. We derived guilds by comparing the cumulative distribution of counts of each species, across all 33,763 count locations, to the cumulative distribution of forest amount in landscapes at each scale using Kolmogorov-Smirnov tests of no difference (see Supplementary Table S10 and Fig. S17). If a large proportion of the individuals of a species occurred in the landscapes with the most forest, then the species was classified as “forest-area sensitive”. Thus, forest-area sensitive species were those that required large amounts of forest in landscapes. Conversely, if a large proportion of the individuals of a species occurred in the landscapes with the least forest, then the species was classified as “edge/open country”. These species required large amounts of non-forest habitat in landscapes. Species that accumulated individuals in landscapes at the same rate that forest cover increased in landscapes were classified as “forest generalists”. Thus, forest generalist species required forest in landscapes but occurred across a broad range of forest amounts, i.e., they were not restricted to landscapes with large amounts of forest as was the case for forest-area sensitive species.

We calculated nine measures of diversity at each count location: relative abundance, species richness, and species evenness for each of the forest-area sensitive, forest generalist, and all forest (area sensitive and generalist species combined) bird guilds. We measured relative abundance as the sum of species counts. We estimated species richness using the Chao1 estimator. Chao1 is a non-parametric estimator of true species richness that is based on the number of rare species observed^69,70^. The estimator performs well when most observations are relatively rare species, as is commonly the case for point counts^69^. We measured species evenness using Pielou’s evenness index^71^. Chao 1 and Pielou’s evenness index were calculated using the fossil^72^ and asbio^73^ packages, respectively, in R, version 3.3.1^74^.

### Forest Amount, Forest Configuration, and Matrix Quality

We measured the amount of forest and its configuration in landscapes using the National Land Cover Database 2006 (NLCD^75^) and FRAGSTATS, version 4.2^76^. We quantified forest amount as the proportional area of landscapes covered by the Deciduous, Evergreen, and Mixed Forest classes. We measured forest configuration using two metrics: patch density and the clumpiness index^76^. We chose these metrics because patch density is one of the more common habitat configuration metrics in the literature, while the clumpiness index is supported as a measure of configuration with low correlation to habitat amount that retains differentiability among landscapes^76–79^. Increasing forest patch density indicates that forest cover in landscapes is divided into a greater number of patches, whereas decreasing forest clumpiness indicates that forest cover in landscapes is more spatially dispersed.

We used degree of urbanization and agriculture amount in landscapes as measures of matrix quality. For each landscape scale, degree of urbanization was derived using a principal component analysis (PCA) of six standardized variables: the proportional area of landscapes in each of the NLCD’s four Developed land covers (Open Space, Low Intensity, Medium Intensity, and High Intensity), area-weighted average population density, and area-weighted average housing density. We measured the population density and housing density variables as the averages of 2010 US Census block-level population and housing densities in landscapes^80^, respectively, weighted by block area^81^. We selected only those principal components that had eigenvalues >1, the Kaiser-Guttman criterion, as meaningful measures of degree of urbanization^82^. The first and second components, accounting for 62–76% of total variance, were selected at the 0.2 and 0.5 km scales while only the first component, accounting for 67–90% of total variance, was selected at larger scales. The first component was highly correlated with population and housing density (*r* > 0.90) and the areas of Developed land covers (*r* ≥ 0.70). The second component was highly correlated with High Intensity Developed cover (*r* ≥ 0.75). We termed the first component simply ‘urbanization’ and the second component ‘high intensity urbanization’. We quantified variation in matrix quality due to agriculture as the proportional area of landscapes covered by the NLCD’s Cultivated Crop and Pasture/Hay land cover classes. We chose the amount of agriculture, rather than a more explicit measure of the intensity of agricultural practices, because, to our knowledge, the latter data do not exist for our study extent. Also, we chose to include both of the NLCD’s agricultural cover classes because they each represented activities that would impact matrix quality for forest birds, such as row cropping (encompassed by the Cultivated Crop class) and pasturing (encompassed by the Pasture class)^83^. Matrix quality variables were created using FRAGSTATS, version 4.2^76^, the isectpolypoly tool in the Geospatial Modelling Environment^81^, and R, version 3.3.1^74^.

### Analyses

We used general linear modeling to estimate the relative effects of forest amount, forest patch density, forest clumpiness index, urbanization, high intensity urbanization, and agriculture amount on each of the nine measures of forest bird diversity. Models also included variables that accounted for local habitat quality, landscape heterogeneity, and species detectability (Table 1; see Supplementary Methods). We standardized all explanatory variables to a mean of 0 and a standard deviation of 1 prior to modeling. We used the negative inverse transformation on the relative abundance and species richness of forest-area sensitive birds and the species richness of forest generalist birds to meet the assumptions of normality and homoscedasticity.

**Table 1:** The explanatory variables included in general linear models of forest bird diversity in Pennsylvania, USA. Landscapes were circular areas surrounding point count locations.

Source of variability	Variable	Description	Data source
Landscape structure	Forest amount	Proportional area of landscapes in Deciduous, Evergreen, and Mixed Forest land covers	National Land Cover Database^75^
Landscape structure	Forest patch density	Number of patches of Deciduous, Evergreen, or Mixed Forest land cover per unit landscape area	National Land Cover Database^75^
Landscape structure	Forest clumpiness index	Spatial aggregation of Deciduous, Evergreen, and Mixed Forest land covers in landscapes	National Land Cover Database^75^
Landscape structure	Urbanization	First component of principal component analysis of the proportional areas of landscapes in Developed land covers and average population and housing densities of landscapes	National Land Cover Database^75^, 2010 US Census^80^
Landscape structure	High intensity urbanization	Second component of principal component analysis of the proportional areas of landscapes in Developed land covers and average population and housing densities of landscapes	National Land Cover Database^75^, 2010 US Census^80^
Landscape structure	Agriculture amount	Proportional area of landscapes in Cultivated Crop and Pasture/Hay land covers	National Land Cover Database^75^
Landscape structure	Landscape heterogeneity 1	First component of principal component analysis of landscape elevation mean and range, Shannon’s diversity of land covers, and forest-developed edge density	3.2-ft digital elevation model of Pennsylvania^87^, National Land Cover Database^75^
Landscape structure	Landscape heterogeneity 2	Second component of principal component analysis of landscape elevation mean and range, Shannon’s diversity of land covers, and forest-developed edge density	3.2-ft digital elevation model of Pennsylvania^87^, National Land Cover Database^75^
Local habitat quality	Land use change	Occurrence of recent or active land use change at point count locations	Second Pennsylvania Breeding Bird Atlas^32^
Local habitat quality	Dominant habitat type	Dominant habitat type within 75 m of count locations	Second Pennsylvania Breeding Bird Atlas^32^
Species detectability	Observer	Observer identity	Second Pennsylvania Breeding Bird Atlas^32^
Species detectability	Start time	Survey start time	Second Pennsylvania Breeding Bird Atlas^32^
Species detectability	Date	Julian date of survey	Second Pennsylvania Breeding Bird Atlas^32^
Species detectability	Year	Survey year	Second Pennsylvania Breeding Bird Atlas^32^

We discerned among the effects of landscape variables measured at different spatial scales using Akaike’s Information Criterion (AIC)^84^. For each measure of forest bird diversity, we ranked models that differed in the scale of measurement of landscape variables, i.e. each model contained landscape variables measured at the same scale, and chose the model with the lowest AIC as the best representation of variation in the forest bird diversity measure.

We chose to use simple general linear modeling to address our research question because it produces unbiased estimates of effect size even when explanatory variables are highly correlated, i.e., *r* = 0.90, assuming that major sources of variation in the response have been included in the model^85^. In our case, the absolute value of correlations between pairs of explanatory variables in the best models of measures of forest bird diversity averaged 0.30 (range = 0.00–0.93) and variance inflation factors averaged 4.40 (range = 1.03–32.97) (see Supplementary Tables S11–S23).

The effects of forest amount, forest configuration, and matrix quality variables estimated in the best model of each measure of forest bird diversity were independent effects, i.e., effects controlling for the effects of all other explanatory variables in the model. For example, the estimated effect of forest amount represented a change in the total amount of forest in a landscape for a given forest patch density, forest clumpiness, degree of urbanization, i.e., housing and population densities and amount of developed cover, amount of agricultural cover, landscape heterogeneity (representing elevation mean and range, Shannon’s diversity of land cover classes, and forest-developed edge density (see Supplementary Methods)), local habitat quality, and species detectability. As such, an increase in forest amount in a landscape occurred at the detriment of open water, barren land, shrubland, herbaceous covers, and/or wetlands, and represented an increase in the amount of habitat available to forest birds. Similarly, a change in forest patch density or forest clumpiness in a landscape represented a change in forest configuration, with no change in the amounts and intensities of other land covers, including forest, landscape heterogeneity, including the amount of forest-developed edge, local habitat quality, or species detectability. An increase in urbanization, high intensity urbanization, or agriculture amount in a landscape represented a positive or negative change in matrix quality for forest birds, depending on whether developed covers, including open space and low-density development, and cultivated crops and pastureland replaced land covers, i.e., open water, barren land, shrubland, herbaceous covers, and/or wetlands, with higher or lower rates of dispersal mortality, fewer or more resources, and/or higher or lower levels of disturbance for forest birds.

In order to gain a general understanding of the relative importance of forest amount, forest configuration, and matrix quality to forest bird diversity overall, we counted the number of times the variable with the largest absolute effect representing each component of landscape structure placed first, second, or third in effect magnitude across the nine diversity measures. In doing so, we considered only meaningful effects, i.e., those with 95% confidence intervals that did not overlap 0, with the exception of meaningless effects when diversity measures were meaningfully affected by two landscape structure components. We ranked these meaningless effects third in relative importance. The landscape structure component that placed first most often across diversity measures was deemed the most important determinant of overall forest bird diversity, the component that placed second most often was considered the second most important determinant, and the component that placed third most often was considered the least important. All analyses were performed in R, version 3.3.1^74^ using the glm function in the stats package and the vif function in the car package^86^.

## Electronic supplementary material

Supplementary Information

## Data Availability

The datasets generated during and/or analysed during the current study are available from the corresponding author on reasonable request.
