# Supplementary material for: The relative effects of forest amount, forest configuration, and urban matrix quality on forest breeding birds
Source: Sci Rep. 2018 Nov 20;8:17140. doi: 10.1038/s41598-018-35276-9 (PMC6244229; doi:10.1038/s41598-018-35276-9)
Supplement: Supplementary file 1 — Supplementary Information [file 41598_2018_35276_MOESM1_ESM.docx]

**The relative effects of forest amount, forest configuration, and urban matrix quality on forest breeding birds**

Alexandra Shoffner, Andrew M. Wilson, Wenwu Tang, and Sara A. Gagné

**Supplementary Methods**

We quantified several explanatory variables in addition to those describing forest amount, forest configuration, and matrix quality. These additional variables can be grouped into measurements of local habitat quality, landscape heterogeneity, and species detectability.

*Local habitat quality*

Ancillary data collected by Pennsylvania Breeding Bird Atlas (PBBA) staff during point counts included two indicators of local habitat quality: evidence of recent or active local land use change (presence or absence of change, such as logging or construction) and the identity of the dominant habitat type, e.g., coniferous forest, deciduous forest, or mixed forest, within 75m of the count location. New or ongoing land use change may deter the occurrence of some bird species who return with time (1) and habitat type affects bird species occurrence (2) by means of variation in niche availability. Local land use change and dominant habitat type may vary independently of the amount and configuration of forest and quality of the matrix in the surrounding landscape.

*Landscape heterogeneity*

For each landscape scale, we created aggregate variables representing landscape heterogeneity using a principal component analysis (PCA) of elevation mean and range, Shannon’s diversity of land cover classes, and forest-developed edge density. These variables encompassed the known effects of topographical variability (3), land cover diversity (4), and forest-developed edges (5) on bird diversity. Land cover diversity represents the diversity of habitats and matrix cover types that may vary independently of habitat amount, habitat configuration, and matrix quality (6-7). Forest-developed edge density quantifies the amount of a particular edge type in a landscape, which is different than the overall amount of edge indirectly measured by forest configuration variables and may vary independently of these, forest amount, and matrix quality. We calculated mean and range of elevation in landscapes using a 3.2-ft digital elevation model from the PAMAP Program (8) and ArcGIS, version 10.3 (9). Using FRAGSTATS, version 4.2 (10), we quantified the Shannon’s diversity of all of the National Land Cover Database (NLCD; 11) classes in landscapes and the density of edges between any of the NLCD's Forest classes and any of its Developed classes. From each PCA conducted at each landscape scale, we selected only those principal components that had eigenvalues > 1, the Kaiser-Guttman criterion, as meaningful measures of landscape heterogeneity (12). The first component (landscape heterogeneity 1) was selected at all scales and the second component (landscape heterogeneity 2) was selected at the 0.2, 0.5, 1, 2, 4, and 6 km scales. The PCA was carried out using R, version 3.3.1 (13).

*Species detectability*

Four variables that directly or indirectly affect the detectability of birds (14-16) were extracted from ancillary data recorded by PBBA staff during point counts: observer identity, survey start time, Julian date, and year.

**References**

1. Gagné, S. A., Sherman, P. J., Singh, K. K. & Meentemeyer, R. K. The effect of human population size on the breeding bird diversity of urban regions. *Biodiversity and Conservation* **25**, 653-671, doi:10.1007/s10531-016-1080-3 (2016).
2. Bergner, A. *et al*. Influences of forest type and habitat structure on bird assemblages of oak (*Quercus* spp.) and pine (*Pinus* spp.) stands in southwestern Turkey. *Forest Ecology and Management* **336**, 137-147 (2015).
3. Davies, R. G. *et al.* Topography, energy and the global distribution of bird species richness. *Proceedings of the Royal Society B* **274**, 1189-1197 (2007).
4. Koivula, M. J. *et al.* Breeding bird species diversity across gradients of land use from forest to agriculture in Europe. *Ecography* **40**, 001-013 (2017).
5. Friesen, L. E., Eagles, P. F. J. & Mackay, R. J. Effects of residential development on forest-dwelling Neotropical migrant songbirds. *Conservation Biology* **9**, 1408-1414 (1995).
6. Fahrig, L. Landscape heterogeneity and metapopulation dynamics in *Key topics in landscape ecology* (eds. Wu, J. & Hobbs, R. J.) 78-91 (Cambridge University Press, 2007).
7. Fahrig, L. *et al.* Functional landscape heterogeneity and animal biodiversity in agricultural landscapes. *Ecology Letters* **14**, 101-112 (2011).
8. Pennsylvania Department of Conservation and Natural Resources. PAMAP program 3.2-ft digital elevation model of Pennsylvania. Dataset at http://www.pasda.psu.edu/uci/DataSummary.aspx?dataset=1247 (2006).
9. ESRI. ArcGIS 10.3 software (ESRI, 2015).
10. McGarigal, K., Cushman, S. A. & Ene, E. FRAGSTATS v4: spatial pattern analysis program for categorical and continuous maps. Computer software program at http://www.umass.edu/landeco/research/fragstats/fragstats.html (2012).
11. Fry, J. *et al.* Completion of the 2006 National Land Cover Database for the conterminous United States. Photogrammetric Engineering & Remote Sensing **77**, 858-864 (2011).
12. Legendre, P. & Legendre, L. Numerical ecology (Elsevier, 1998).
13. R Core Team. R: a language and environment for statistical computing. Computer software program at https://www.R-project.org (2016).
14. Farnsworth, G. L. *et al.* A removal model for estimating detection probabilities from point-count surveys. *The Auk* **119**, 414-425 (2002).
15. Link, W. A. & Sauer, J. R. A hierarchical analysis of population change with application to cerulean warblers. *Ecology* **83**, 2832-2840 (2002).
16. Furnas, B. J. & Callas, R. L. Using automated recorders and occupancy models to monitor common forest birds across a large geographic region. *The Journal of Wildlife Management* **79**, 325-337 (2015).

**Supplementary Figures**


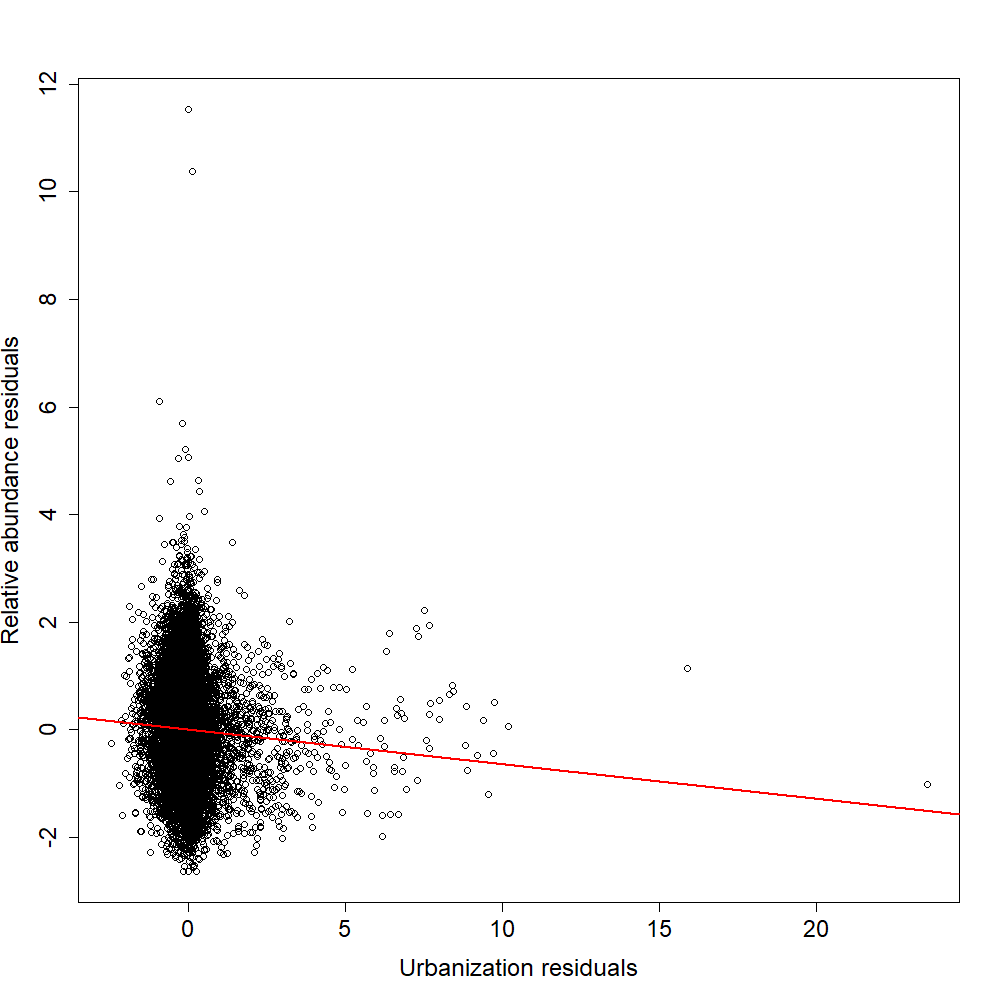


**Figure S1.** The effect of urbanization on the relative abundance of all forest birds in Pennsylvania, USA. Relative abundance residuals are the product of a model regressing relative abundance against all explanatory variables except urbanization. Urbanization residuals are the product of a model regressing urbanization against all other explanatory variables. Landscape variables in models were measured at the 0.2 km scale.


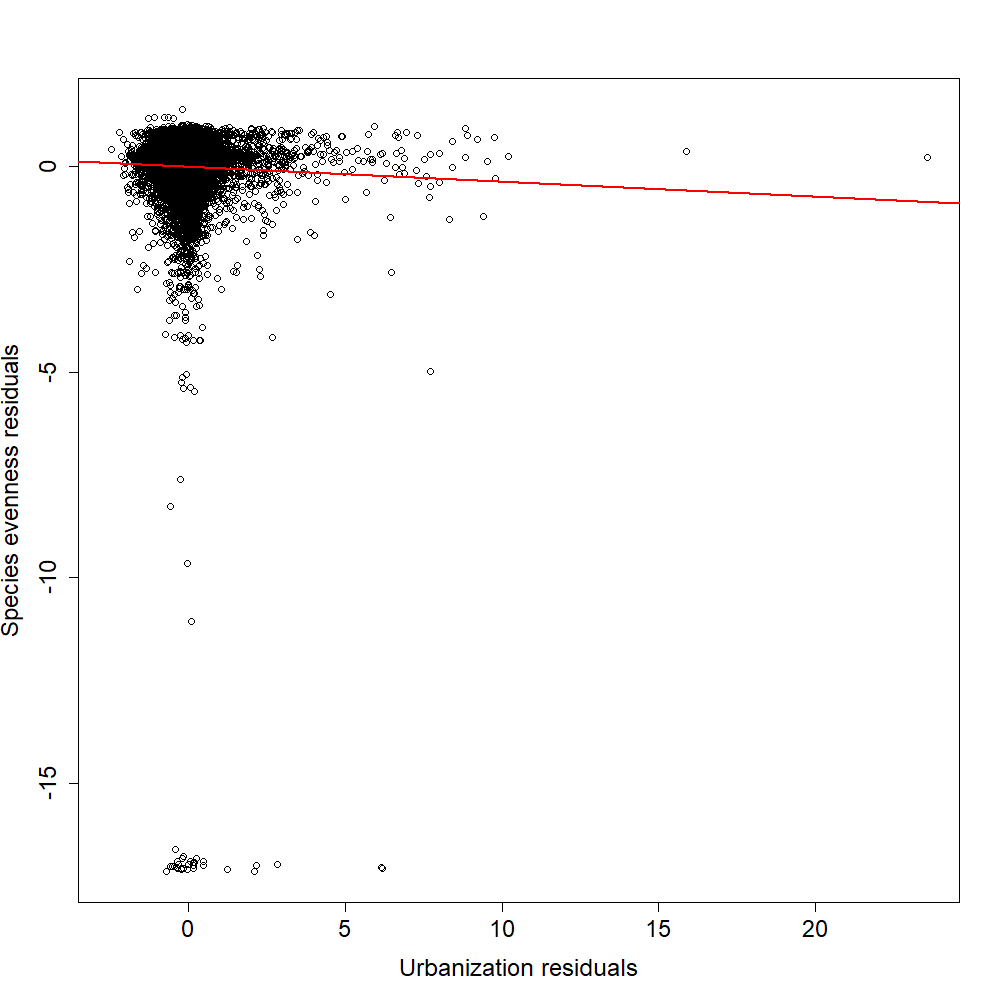


**Figure S2.** The effect of urbanization on the species evenness of all forest birds in Pennsylvania, USA. Species evenness residuals are the product of a model regressing species evenness against all explanatory variables except urbanization. Urbanization residuals are the product of a model regressing urbanization against all other explanatory variables. Landscape variables in models were measured at the 0.2 km scale.


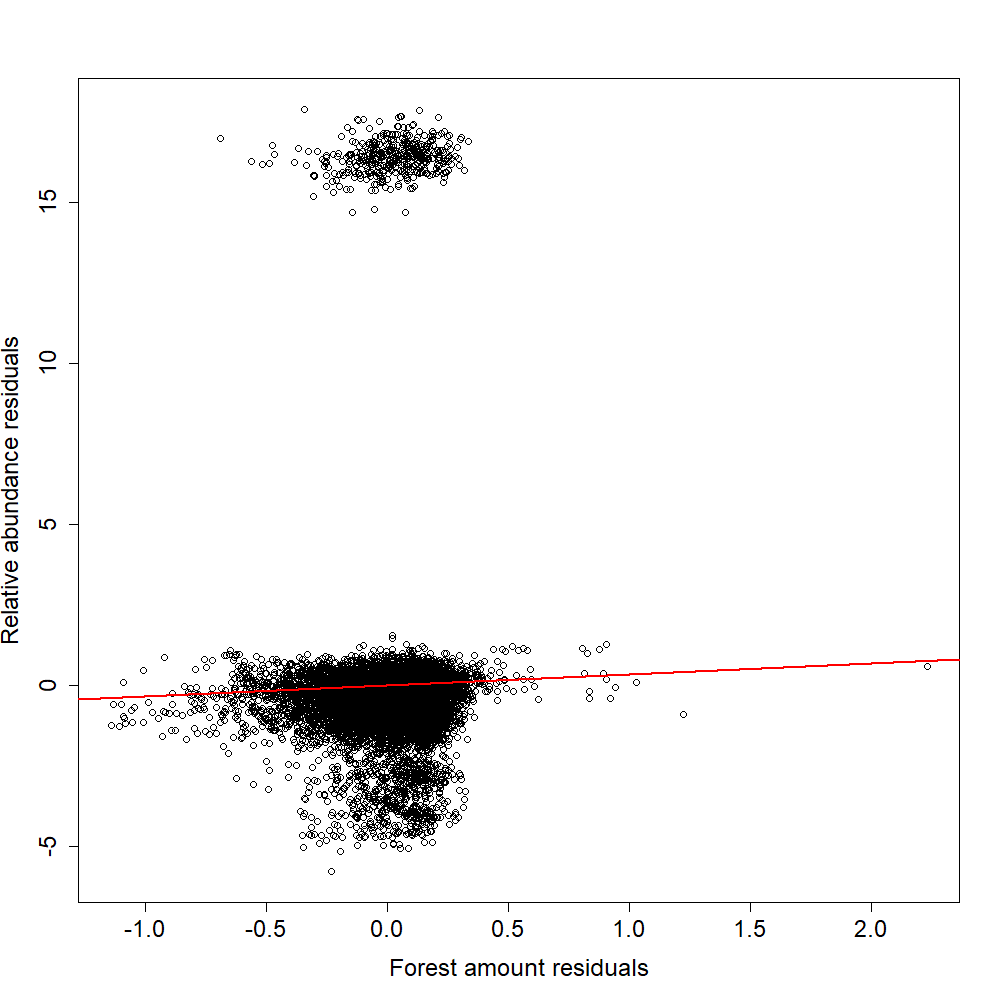


**Figure S3.** The effect of forest amount on the relative abundance of forest-area sensitive birds in Pennsylvania, USA. Relative abundance residuals are the product of a model regressing relative abundance against all explanatory variables except forest amount. Forest amount residuals are the product of a model regressing forest amount against all other explanatory variables. Landscape variables in models were measured at the 6 km scale.


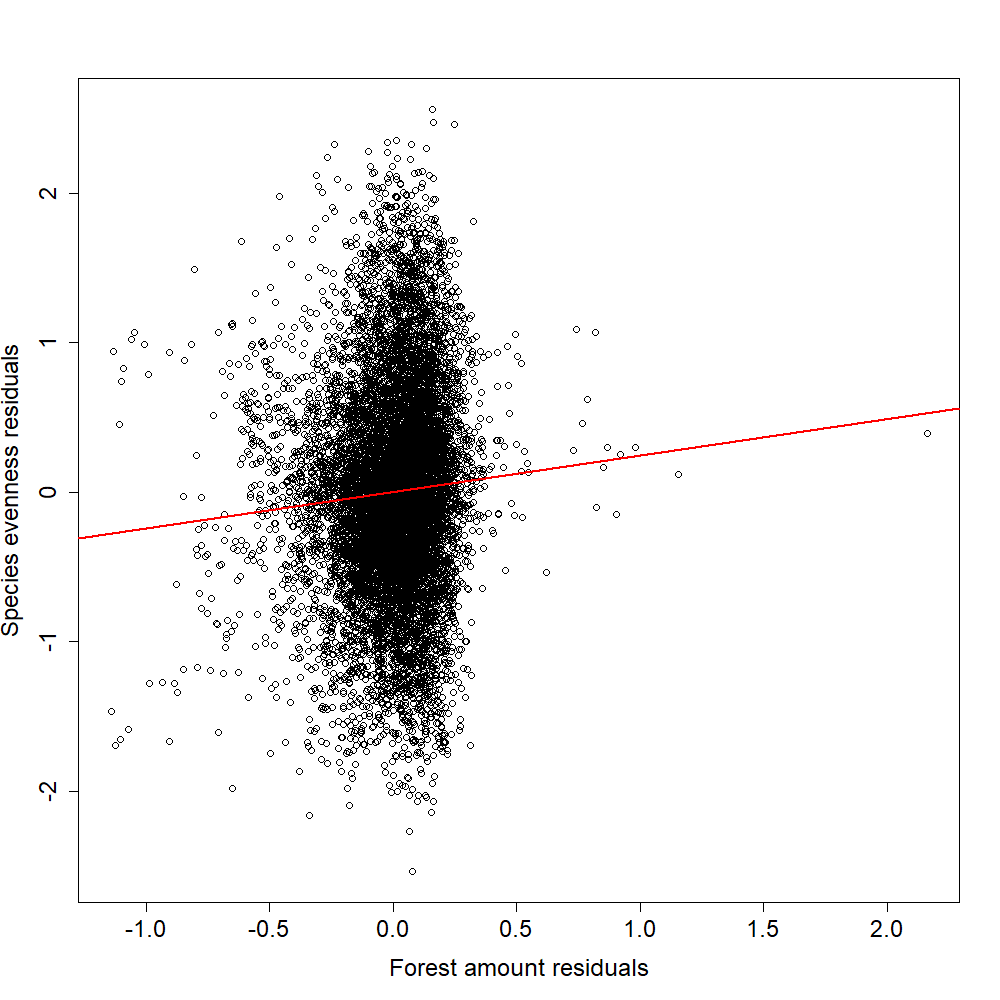


**Figure S4.** The effect of forest amount on the species evenness of forest-area sensitive birds in Pennsylvania, USA. Species evenness residuals are the product of a model regressing species evenness against all explanatory variables except forest amount. Forest amount residuals are the product of a model regressing forest amount against all other explanatory variables. Landscape variables in models were measured at the 6 km scale.


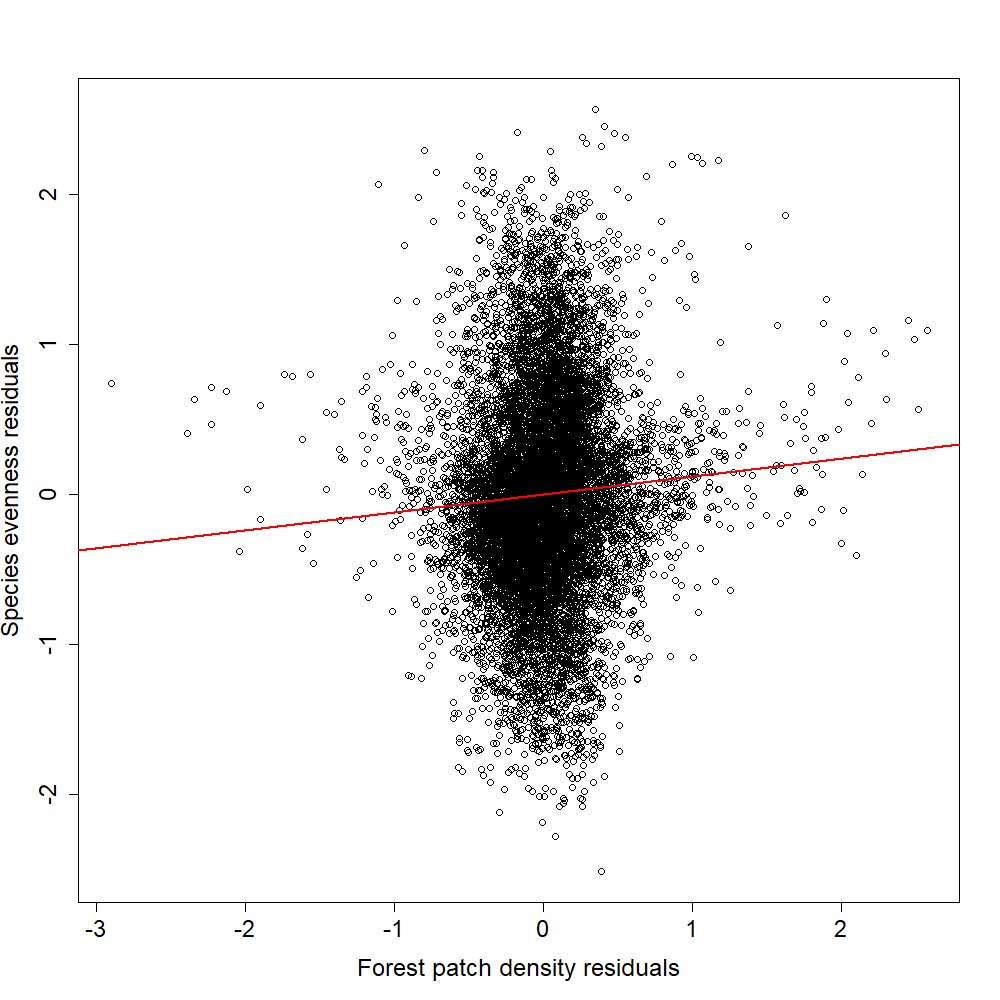


**Figure S5.** The effect of forest patch density on the species evenness of forest-area sensitive birds in Pennsylvania, USA. Species evenness residuals are the product of a model regressing species evenness against all explanatory variables except forest patch density. Forest patch density residuals are the product of a model regressing forest patch density against all other explanatory variables. Landscape variables in models were measured at the 6 km scale.


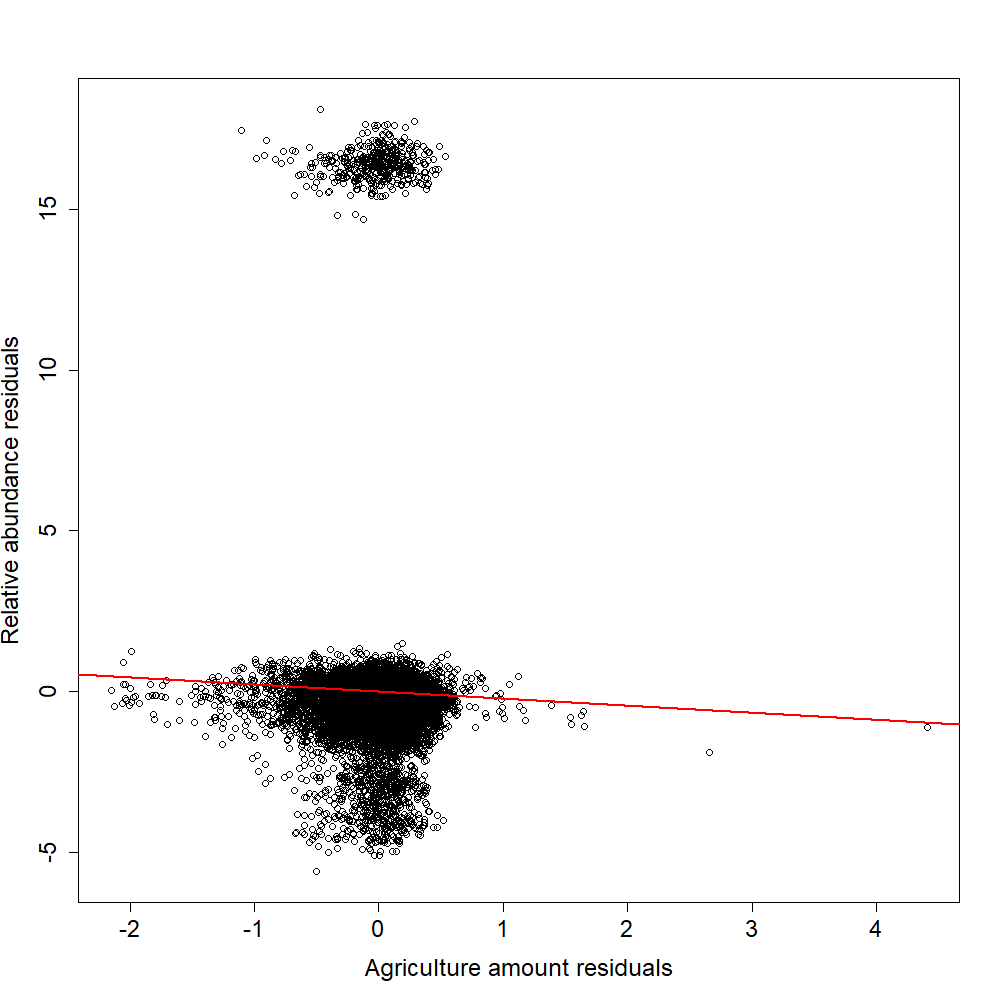


**Figure S6.** The effect of agriculture amount on the relative abundance of forest-area sensitive birds in Pennsylvania, USA. Relative abundance residuals are the product of a model regressing relative abundance against all explanatory variables except agriculture amount. Agriculture amount residuals are the product of a model regressing agriculture amount against all other explanatory variables. Landscape variables in models were measured at the 6 km scale.


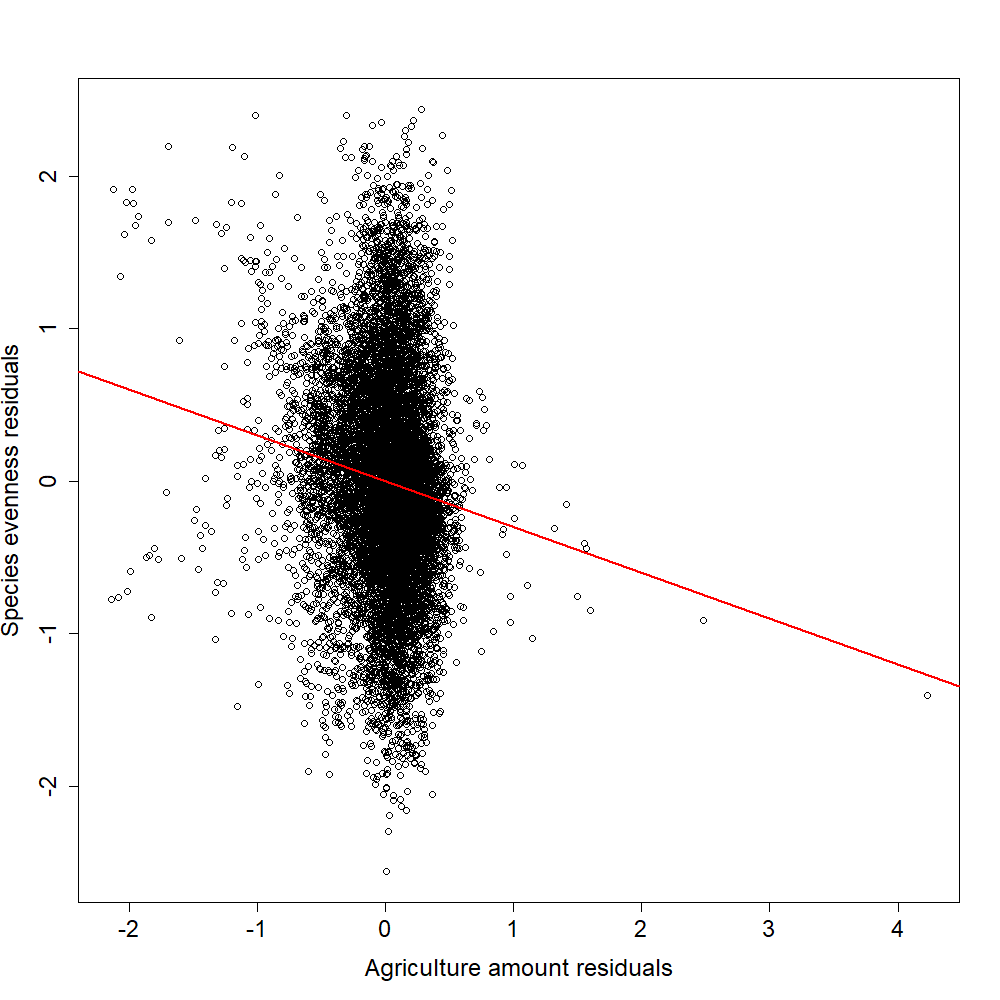


**Figure S7.** The effect of agriculture amount on the species evenness of forest-area sensitive birds in Pennsylvania, USA. Species evenness residuals are the product of a model regressing species evenness against all explanatory variables except agriculture amount. Agriculture amount residuals are the product of a model regressing agriculture amount against all other explanatory variables. Landscape variables in models were measured at the 6 km scale.


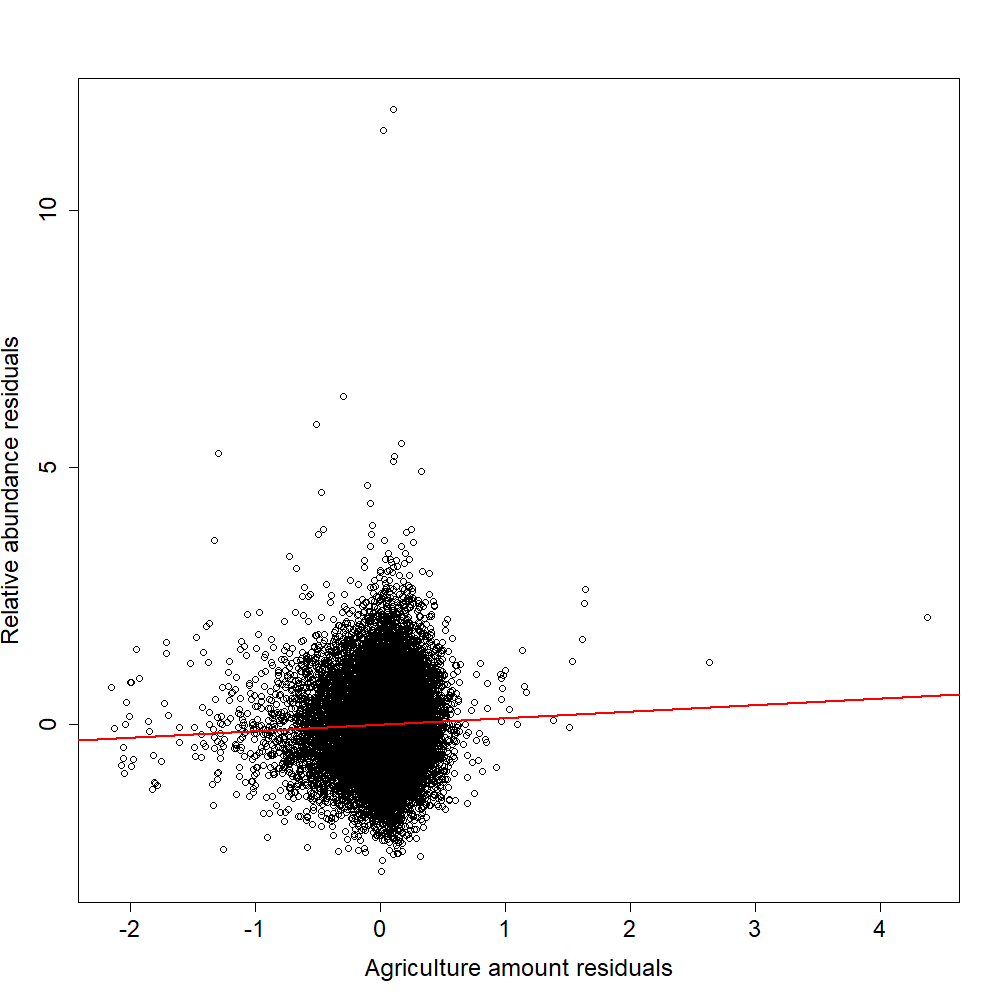


**Figure S8.** The effect of agriculture amount on the relative abundance of forest generalist birds in Pennsylvania, USA. Relative abundance residuals are the product of a model regressing relative abundance against all explanatory variables except agriculture amount. Agriculture amount residuals are the product of a model regressing agriculture amount against all other explanatory variables. Landscape variables in models were measured at the 6 km scale.


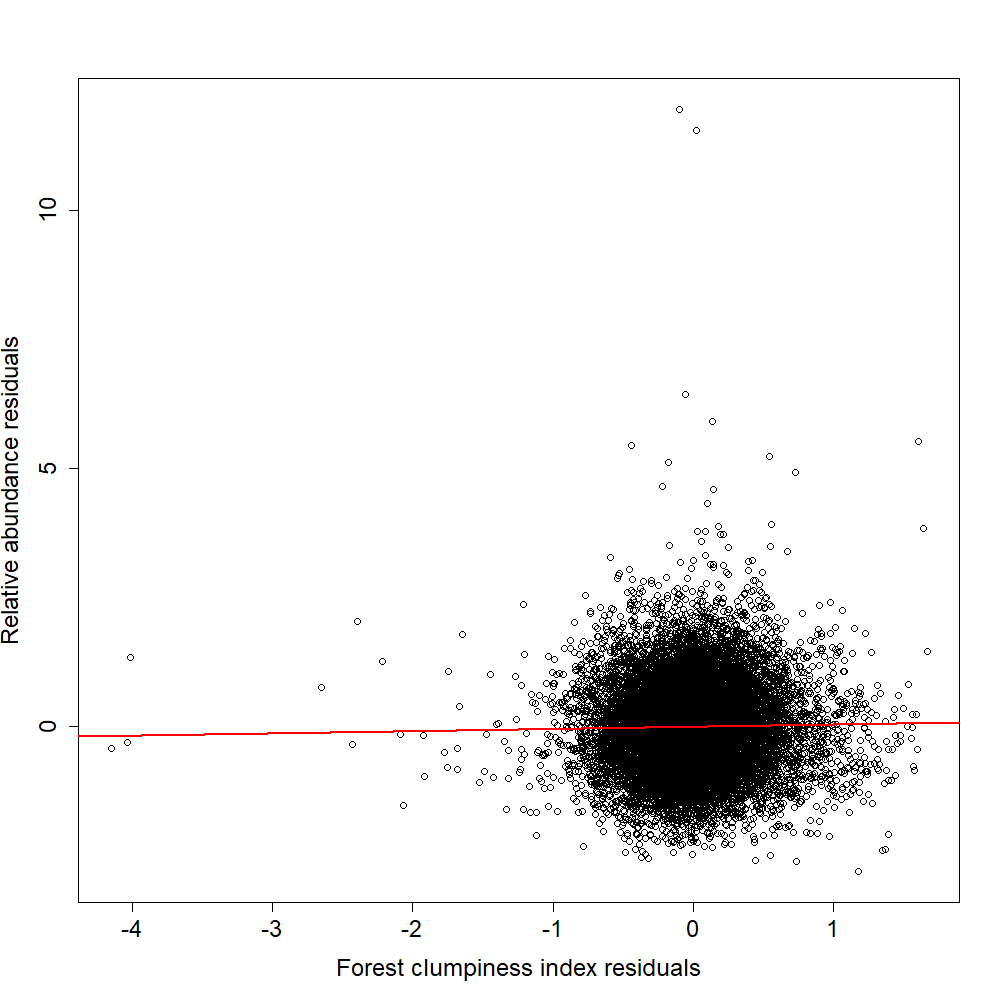


**Figure S9.** The effect of forest clumpiness index on the relative abundance of forest generalist birds in Pennsylvania, USA. Relative abundance residuals are the product of a model regressing relative abundance against all explanatory variables except forest clumpiness index. Forest clumpiness index residuals are the product of a model regressing forest clumpiness index against all other explanatory variables. Landscape variables in models were measured at the 6 km scale.


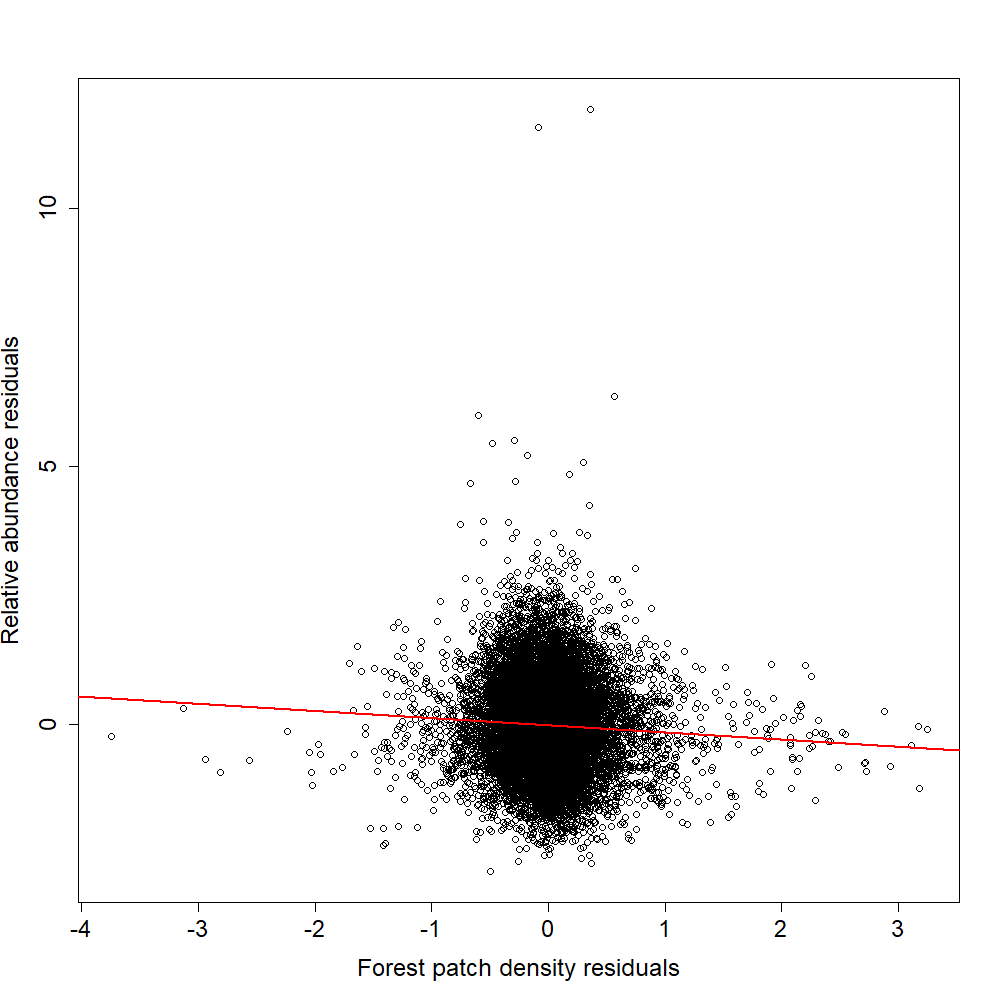


**Figure S10.** The effect of forest patch density on the relative abundance of forest generalist birds in Pennsylvania, USA. Relative abundance residuals are the product of a model regressing relative abundance against all explanatory variables except forest patch density. Forest patch density residuals are the product of a model regressing forest patch density against all other explanatory variables. Landscape variables in models were measured at the 6 km scale.


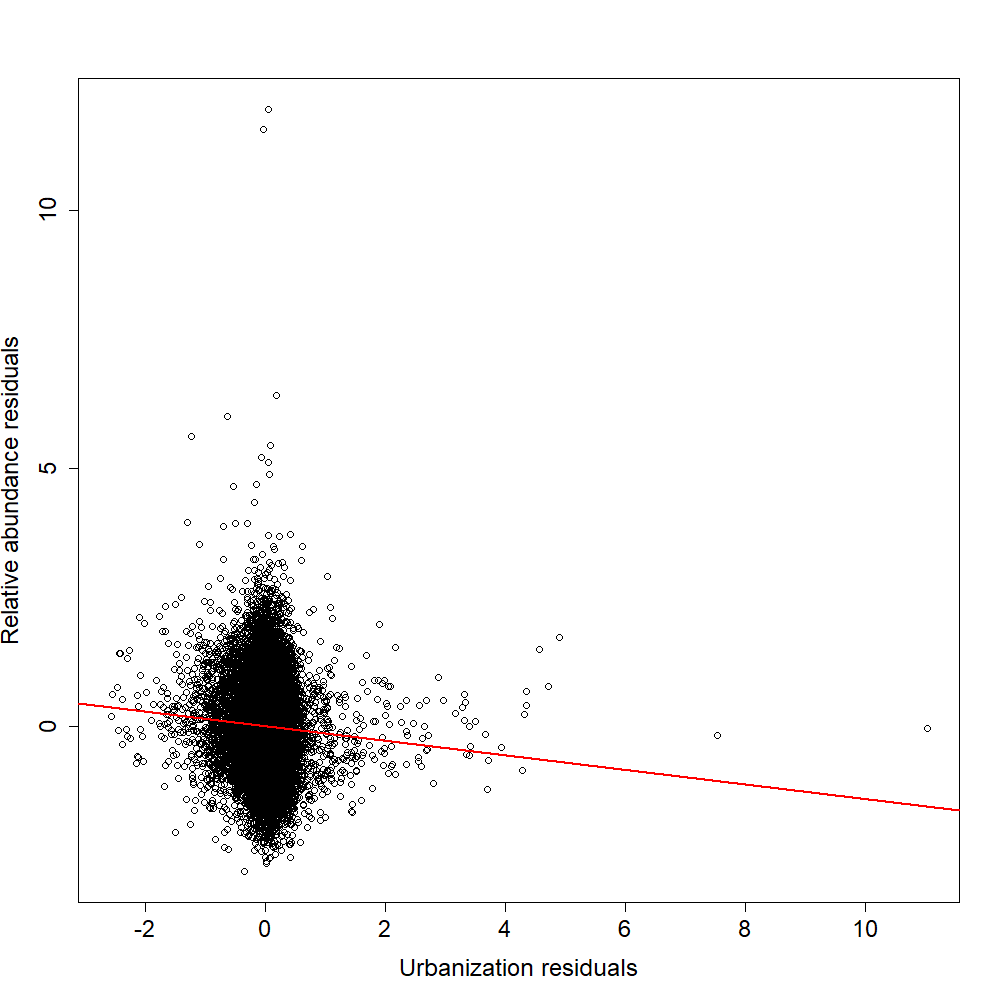


**Figure S11.** The effect of urbanization on the relative abundance of forest generalist birds in Pennsylvania, USA. Relative abundance residuals are the product of a model regressing relative abundance against all explanatory variables except urbanization. Urbanization residuals are the product of a model regressing urbanization against all other explanatory variables. Landscape variables in models were measured at the 6 km scale.


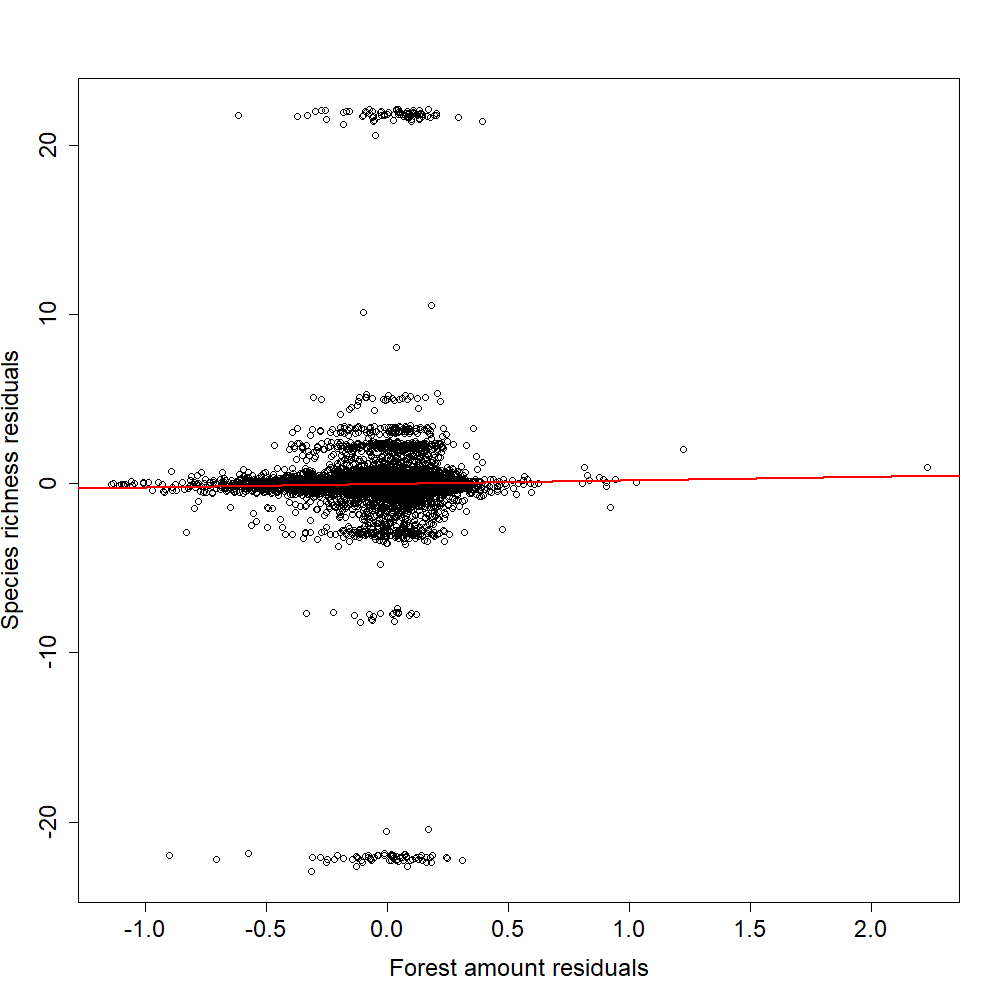


**Figure S12.** The effect of forest amount on the species richness of forest generalist birds in Pennsylvania, USA. Species richness residuals are the product of a model regressing species richness against all explanatory variables except forest amount. Forest amount residuals are the product of a model regressing forest amount against all other explanatory variables. Landscape variables in models were measured at the 10 km scale.


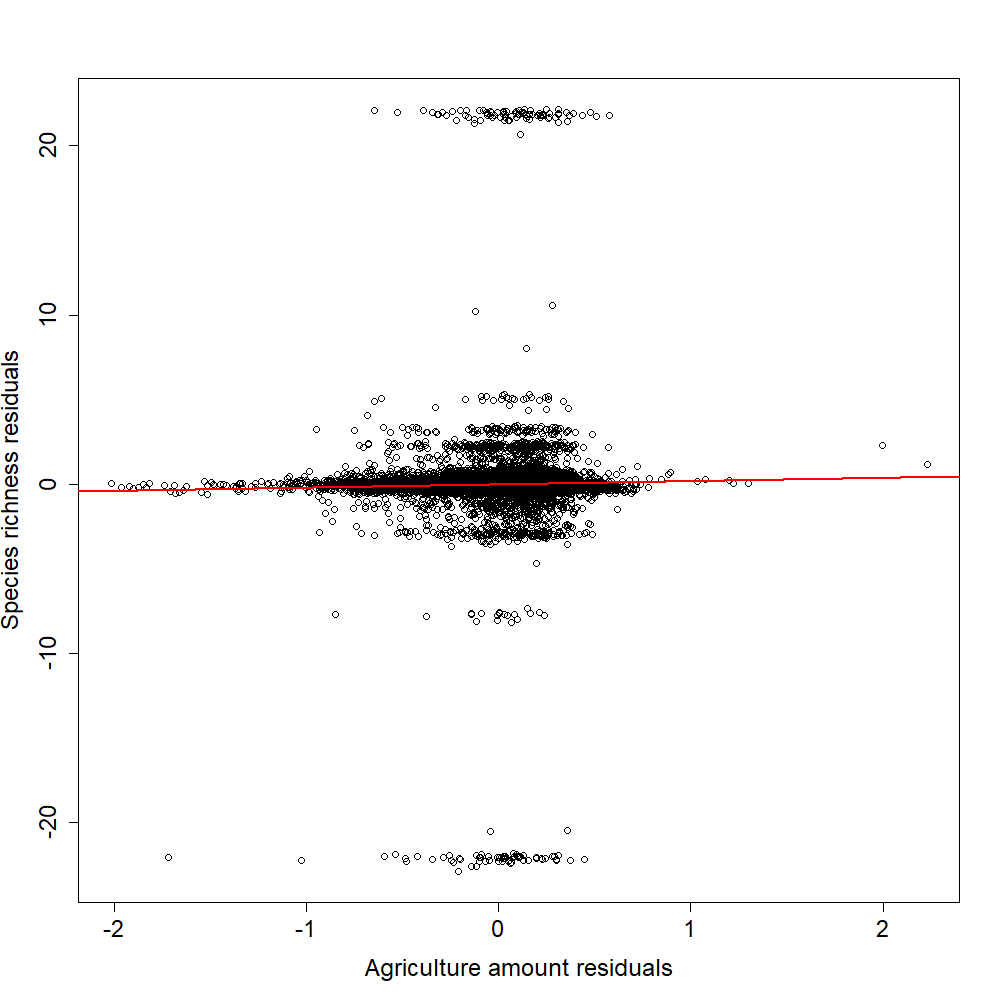


**Figure S13.** The effect of agriculture amount on the species richness of forest generalist birds in Pennsylvania, USA. Species richness residuals are the product of a model regressing species richness against all explanatory variables except agriculture amount. Agriculture amount residuals are the product of a model regressing agriculture amount against all other explanatory variables. Landscape variables in models were measured at the 10 km scale.


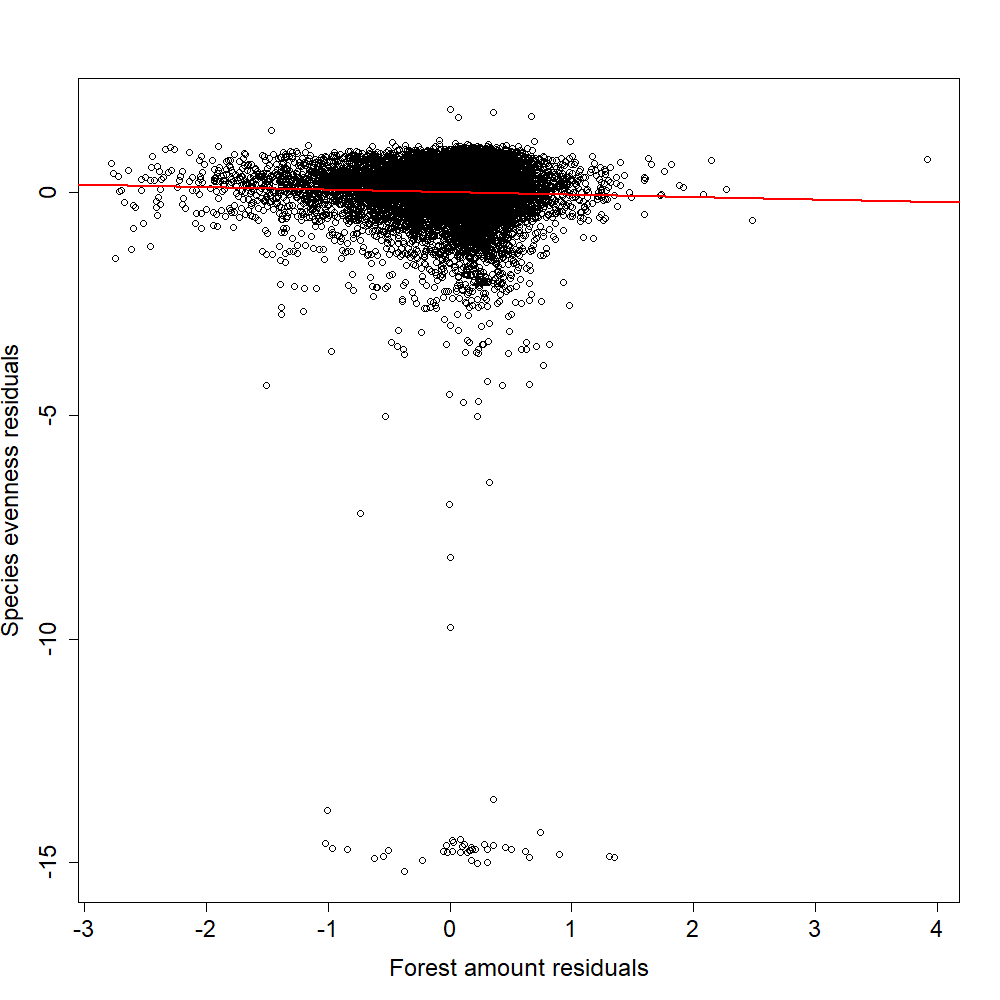


**Figure S14.** The effect of forest amount on the species evenness of forest generalist birds in Pennsylvania, USA. Species evenness residuals are the product of a model regressing species evenness against all explanatory variables except forest amount. Forest amount residuals are the product of a model regressing forest amount against all other explanatory variables. Landscape variables in models were measured at the 0.2 km scale.


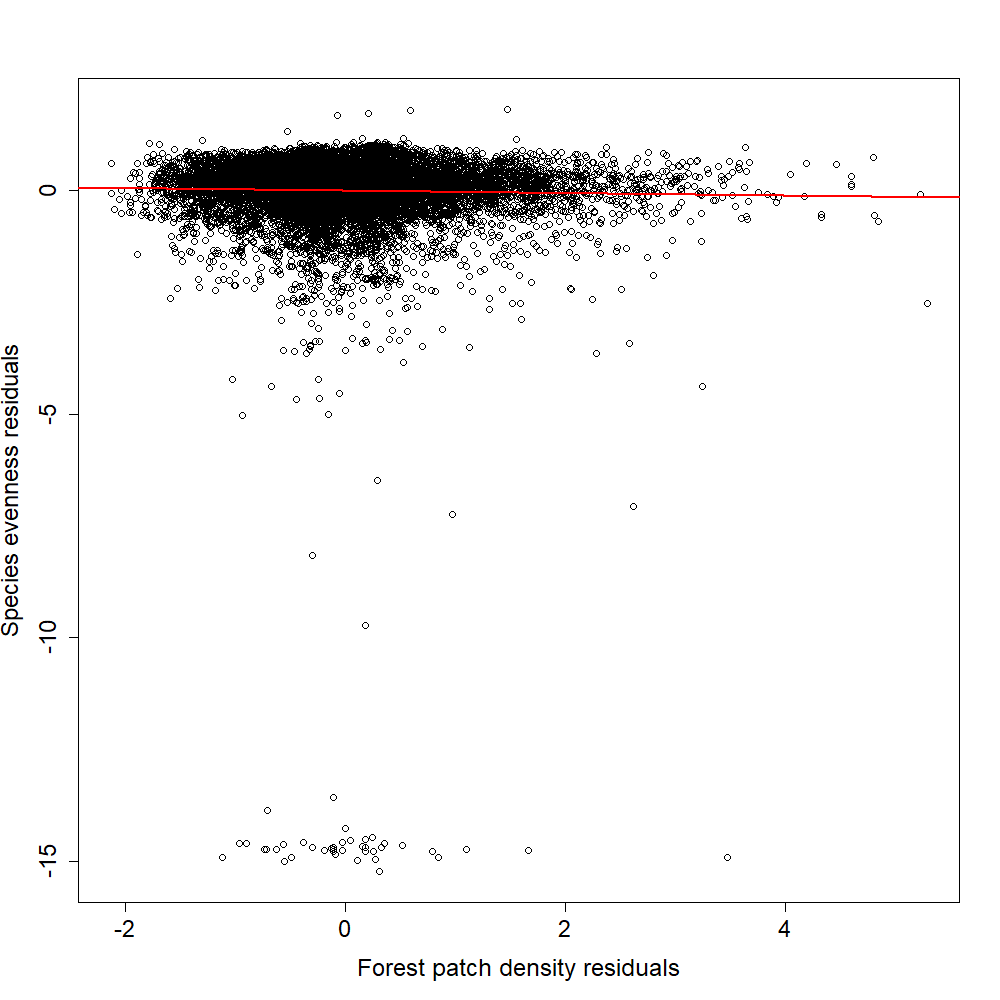


**Figure S15.** The effect of forest patch density on the species evenness of forest generalist birds in Pennsylvania, USA. Species evenness residuals are the product of a model regressing species evenness against all explanatory variables except forest patch density. Forest patch density residuals are the product of a model regressing forest patch density against all other explanatory variables. Landscape variables in models were measured at the 0.2 km scale.


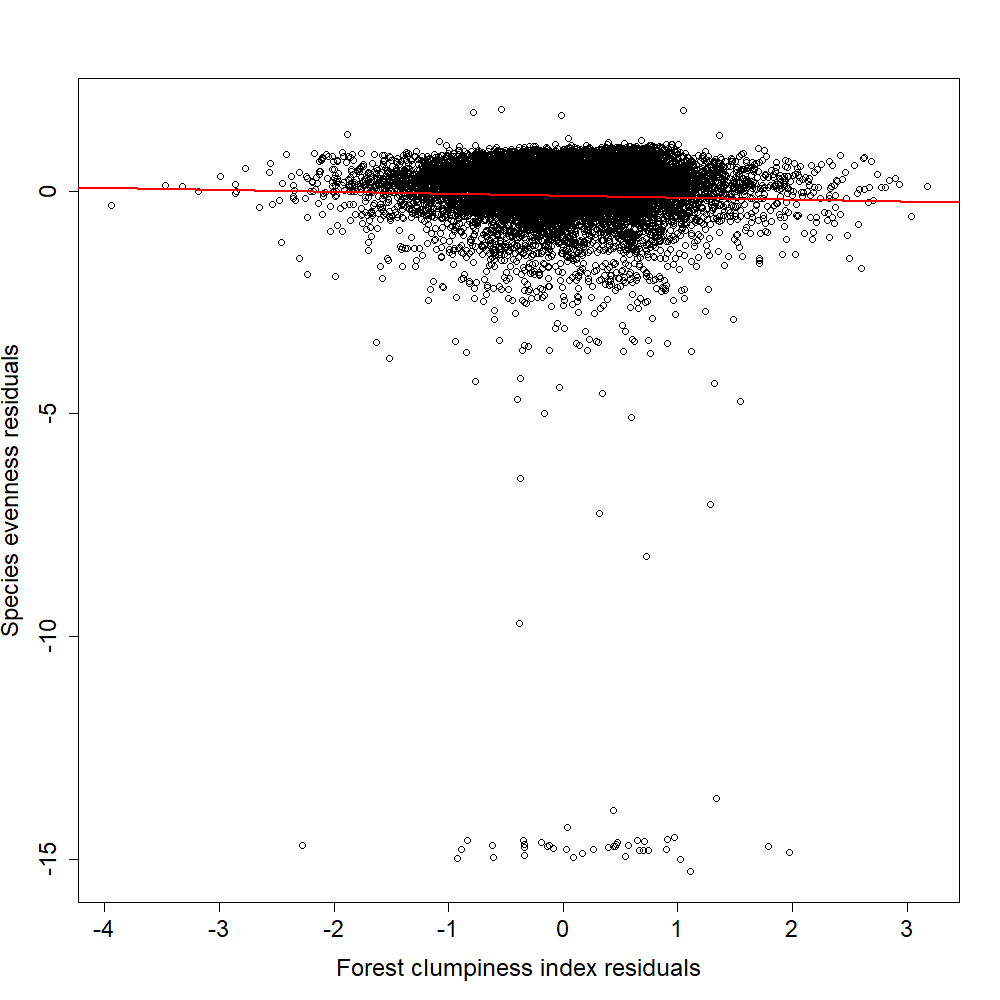


**Figure S16.** The effect of forest clumpiness index on the species evenness of forest generalist birds in Pennsylvania, USA. Species evenness residuals are the product of a model regressing species evenness against all explanatory variables except forest clumpiness index. Forest clumpiness index residuals are the product of a model regressing forest clumpiness index against all other explanatory variables. Landscape variables in models were measured at the 0.2 km scale.


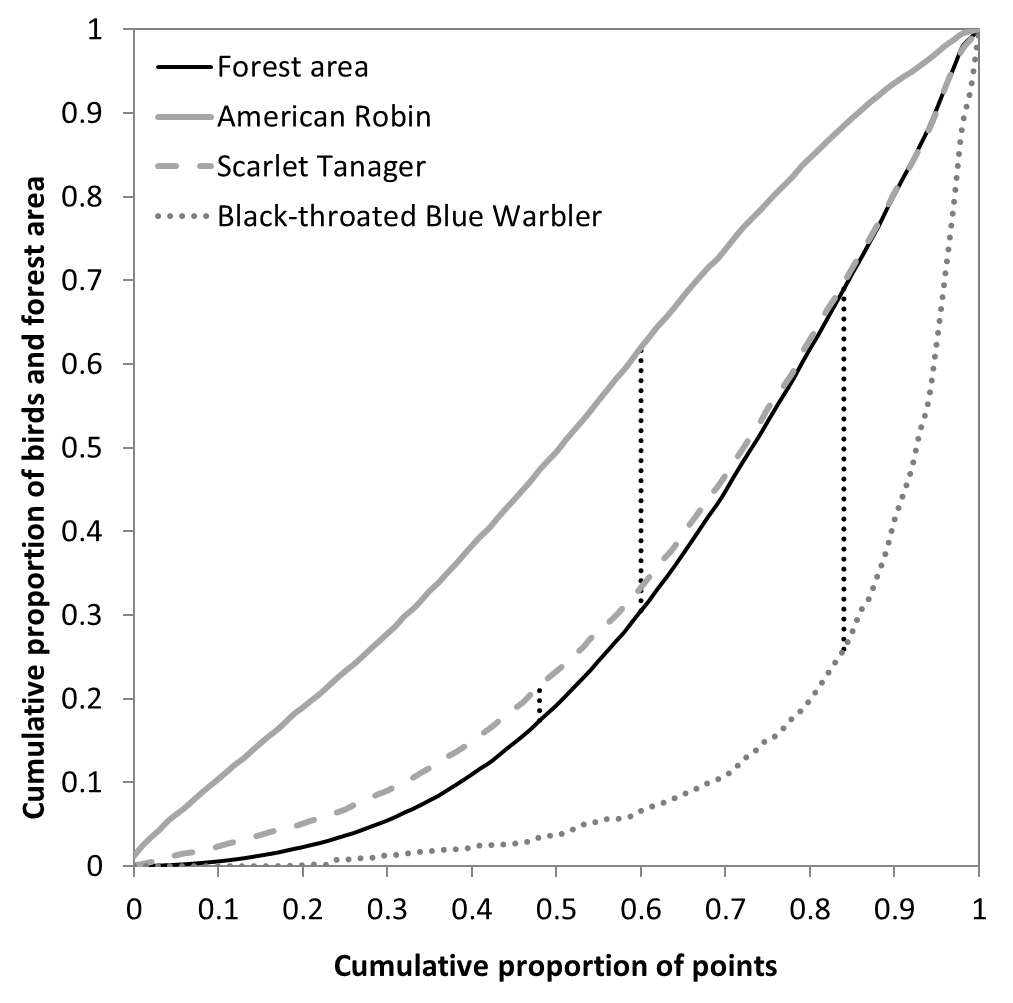


**Figure S17.** Example of classification of species into habitat association guilds based on the cumulative distribution of their counts and the cumulative distribution of forest cover in landscapes surrounding count locations (N = 33,763). This example is for landscapes of 1-km radius. Dotted black lines represent the largest deviances between the bird count curve and the forest cover curve, as measured by *D*, the Kolmogorov-Smirnov (KS) test statistic. The null hypothesis in these tests is that the bird count and forest cover distributions are identical. In other words, the cumulative increase in bird counts in landscapes occurs at the same rate as the cumulative increase in forest cover in landscapes. If this is the case, then the species in question increased in abundance in proportion to the amount of forest in landscapes.

The Black-throated Blue Warbler's (*Setophaga caerulescens*) count distribution differed significantly from that of forest cover (*D* = 0.43, *p* < 0.001). In this case, the top 16% most forested landscapes encompassed 31% of all forest cover but accounted for 74% of birds, indicating that this species preferred landscapes with large amounts of forest. The American Robin's (*Turdus migratorius*) count distribution also differed significantly from that of forest cover (*D* = -0.31, *p* < 0.001) but a large proportion of American Robins occurred in landscapes with less forest, indicating that this species required large amounts of non-forest habitat in landscapes. The Scarlet Tanager's (*Piranga olivacea*) count distribution did not differ significantly from that of forest cover (*D* = 0.04, *p* = 0.851), indicating that this species occurred in proportion to forest cover in landscapes. In other words, the Scarlet Tanager required forest in landscapes but occurred across a broad range of forest amounts, i.e., it was not restricted to landscapes with large amounts of forest as was the Black-throated Blue Warbler.

Species were assigned to one of three habitat association guilds based on the results of KS tests at 10 landscape scales (Table S13). In the cases of the above three species, the Black-throated Blue Warbler's results at all 10 scales indicated that it preferred landscapes with large amounts of forest, and hence the species was classified as “forest-area sensitive”. The American Robin was found disproportionately in less forested landscapes at all 10 scales, and hence was classified as “edge/open country”. The Scarlet Tanager was found in proportion to forest cover at all 10 scales, and hence was classified as “forest generalist”. In general, species were classified into forest-area sensitive and edge/open country guilds based on whether the majority of their test statistic values across scales were significantly positive or significantly negative, respectively. Species that did not meet either of these criteria were classified as forest generalists. We chose this method of assignation because we had no a priori knowledge of the scales at which landscape variables would affect measures of forest bird diversity.

**Supplementary Tables**

**Supplementary Table S1.** The performance of general linear models describing the relative abundance of all forest birds in Pennsylvania, USA and containing landscape variables measured at different spatial scales. All models included the explanatory variables forest amount, forest patch density, forest clumpiness index, urbanization, agriculture amount, landscape heterogeneity 1, land use change, dominant habitat type, observer, start time, date, and year (see Table 1 for variable descriptions). Models at the 0.2 and 0.5 km scales also included the variable high intensity urbanization and models at the 0.2, 0.5, 1, 2, 4, and 6 km scales also included the variable landscape heterogeneity 2. K = the number of estimated parameters; AIC = Akaike's Information Criterion; $\Delta_{i}$ = AIC*_i_* – minAIC for each model *i*; w*_i_* = Akaike weight, or probability of being the best model given the observed data and the set of models evaluated.

| Scale (km) | AIC | K | $\Delta_{i}$ | w*_i_* |
| --- | --- | --- | --- | --- |
| 0.2 | 38030.33 | 57 | 0.00 | 1.00 |
| 0.5 | 38134.72 | 57 | 104.39 | 0.00 |
| 2 | 38345.52 | 56 | 315.19 | 0.00 |
| 1 | 38353.35 | 56 | 323.02 | 0.00 |
| 4 | 38429.65 | 56 | 399.32 | 0.00 |
| 8 | 38528.30 | 55 | 497.97 | 0.00 |
| 6 | 38549.10 | 56 | 518.77 | 0.00 |
| 16 | 38580.48 | 55 | 550.15 | 0.00 |
| 12 | 38705.41 | 55 | 675.08 | 0.00 |
| 10 | 38735.07 | 55 | 704.74 | 0.00 |

**Supplementary Table S2.** The performance of general linear models describing the species richness of all forest birds in Pennsylvania, USA and containing landscape variables measured at different spatial scales. All models included the explanatory variables forest amount, forest patch density, forest clumpiness index, urbanization, agriculture amount, landscape heterogeneity 1, land use change, dominant habitat type, observer, start time, date, and year (see Table 1 for variable descriptions). Models at the 0.2 and 0.5 km scales also included the variable high intensity urbanization and models at the 0.2, 0.5, 1, 2, 4, and 6 km scales also included the variable landscape heterogeneity 2. K = the number of estimated parameters; AIC = Akaike's Information Criterion; $\Delta_{i}$ = AIC*_i_* – minAIC for each model *i*; w*_i_* = Akaike weight, or probability of being the best model given the observed data and the set of models evaluated.

| Scale (km) | AIC | K | $\Delta_{i}$ | w*_i_* |
| --- | --- | --- | --- | --- |
| 0.2 | 38856.07 | 57 | 0.00 | 1.00 |
| 0.5 | 38926.03 | 57 | 69.96 | 0.00 |
| 1 | 38950.25 | 56 | 94.18 | 0.00 |
| 2 | 38964.55 | 56 | 108.48 | 0.00 |
| 4 | 38991.48 | 56 | 135.41 | 0.00 |
| 8 | 38992.28 | 55 | 136.21 | 0.00 |
| 6 | 39021.69 | 56 | 165.62 | 0.00 |
| 12 | 39021.96 | 55 | 165.89 | 0.00 |
| 10 | 39027.55 | 55 | 171.48 | 0.00 |
| 16 | 39046.36 | 55 | 190.29 | 0.00 |

**Supplementary Table S3.** The performance of general linear models describing the species evenness of all forest birds in Pennsylvania, USA and containing landscape variables measured at different spatial scales. All models included the explanatory variables forest amount, forest patch density, forest clumpiness index, urbanization, agriculture amount, landscape heterogeneity 1, land use change, dominant habitat type, observer, start time, date, and year (see Table 1 for variable descriptions). Models at the 0.2 and 0.5 km scales also included the variable high intensity urbanization and models at the 0.2, 0.5, 1, 2, 4, and 6 km scales also included the variable landscape heterogeneity 2. K = the number of estimated parameters; AIC = Akaike's Information Criterion; $\Delta_{i}$ = AIC*_i_* – minAIC for each model *i*; w*_i_* = Akaike weight, or probability of being the best model given the observed data and the set of models evaluated.

| Scale (km) | AIC | K | $\Delta_{i}$ | w*_i_* |
| --- | --- | --- | --- | --- |
| 0.2 | 38739.53 | 57 | 0.00 | 1.00 |
| 8 | 38780.66 | 55 | 41.13 | 0.00 |
| 0.5 | 38780.84 | 57 | 41.31 | 0.00 |
| 1 | 38798.75 | 56 | 59.22 | 0.00 |
| 2 | 38799.32 | 56 | 59.79 | 0.00 |
| 12 | 38800.75 | 55 | 61.22 | 0.00 |
| 6 | 38801.73 | 56 | 62.20 | 0.00 |
| 4 | 38807.89 | 56 | 68.36 | 0.00 |
| 16 | 38825.39 | 55 | 85.86 | 0.00 |
| 10 | 38841.21 | 55 | 101.68 | 0.00 |

**Supplementary Table S4.** The performance of general linear models describing the relative abundance of forest-area sensitive birds in Pennsylvania, USA and containing landscape variables measured at different spatial scales. All models included the explanatory variables forest amount, forest patch density, forest clumpiness index, urbanization, agriculture amount, landscape heterogeneity 1, land use change, dominant habitat type, observer, start time, date, and year (see Table 1 for variable descriptions). Models at the 0.2 and 0.5 km scales also included the variable high intensity urbanization and models at the 0.2, 0.5, 1, 2, 4, and 6 km scales also included the variable landscape heterogeneity 2. K = the number of estimated parameters; AIC = Akaike's Information Criterion; $\Delta_{i}$ = AIC*_i_* – minAIC for each model *i*; w*_i_* = Akaike weight, or probability of being the best model given the observed data and the set of models evaluated.

| Scale (km) | AIC | K | $\Delta_{i}$ | w*_i_* |
| --- | --- | --- | --- | --- |
| 6 | 69679.33 | 56 | 0.00 | 1.00 |
| 2 | 69695.56 | 56 | 16.23 | 0.00 |
| 4 | 69697.18 | 56 | 17.85 | 0.00 |
| 1 | 69713.17 | 56 | 33.84 | 0.00 |
| 0.5 | 69719.25 | 57 | 39.92 | 0.00 |
| 0.2 | 69749.48 | 57 | 70.15 | 0.00 |
| 10 | 69755.70 | 55 | 76.37 | 0.00 |
| 8 | 69757.84 | 55 | 78.51 | 0.00 |
| 12 | 69758.39 | 55 | 79.06 | 0.00 |
| 16 | 69767.05 | 55 | 87.72 | 0.00 |

**Supplementary Table S5.** The performance of general linear models describing the species richness of forest-area sensitive birds in Pennsylvania, USA and containing landscape variables measured at different spatial scales. All models included the explanatory variables forest amount, forest patch density, forest clumpiness index, urbanization, agriculture amount, landscape heterogeneity 1, land use change, dominant habitat type, observer, start time, date, and year (see Table 1 for variable descriptions). Models at the 0.2 and 0.5 km scales also included the variable high intensity urbanization and models at the 0.2, 0.5, 1, 2, 4, and 6 km scales also included the variable landscape heterogeneity 2. K = the number of estimated parameters; AIC = Akaike's Information Criterion; $\Delta_{i}$ = AIC*_i_* – minAIC for each model *i*; w*_i_* = Akaike weight, or probability of being the best model given the observed data and the set of models evaluated.

| Scale (km) | AIC | K | $\Delta_{i}$ | w*_i_* |
| --- | --- | --- | --- | --- |
| 1 | 87517.93 | 56 | 0.00 | 0.56 |
| 4 | 87519.81 | 56 | 1.88 | 0.22 |
| 2 | 87521.49 | 56 | 3.56 | 0.10 |
| 6 | 87523.31 | 56 | 5.38 | 0.04 |
| 0.5 | 87523.57 | 57 | 5.64 | 0.03 |
| 8 | 87525.85 | 55 | 7.92 | 0.01 |
| 10 | 87525.88 | 55 | 7.95 | 0.01 |
| 12 | 87525.91 | 55 | 7.98 | 0.01 |
| 0.2 | 87526.27 | 57 | 8.34 | 0.01 |
| 16 | 87526.56 | 55 | 8.63 | 0.01 |

**Supplementary Table S6.** The performance of general linear models describing the species evenness of forest-area sensitive birds in Pennsylvania, USA and containing landscape variables measured at different spatial scales. All models included the explanatory variables forest amount, forest patch density, forest clumpiness index, urbanization, agriculture amount, landscape heterogeneity 1, land use change, dominant habitat type, observer, start time, date, and year (see Table 1 for variable descriptions). Models at the 0.2 and 0.5 km scales also included the variable high intensity urbanization and models at the 0.2, 0.5, 1, 2, 4, and 6 km scales also included the variable landscape heterogeneity 2. K = the number of estimated parameters; AIC = Akaike's Information Criterion; $\Delta_{i}$ = AIC*_i_* – minAIC for each model *i*; w*_i_* = Akaike weight, or probability of being the best model given the observed data and the set of models evaluated.

| Scale (km) | AIC | K | $\Delta_{i}$ | w*_i_* |
| --- | --- | --- | --- | --- |
| 6 | 23914.07 | 56 | 0.00 | 1.00 |
| 2 | 24232.79 | 56 | 318.72 | 0.00 |
| 4 | 24269.77 | 56 | 355.70 | 0.00 |
| 1 | 24706.20 | 56 | 792.13 | 0.00 |
| 16 | 25051.50 | 55 | 1137.43 | 0.00 |
| 12 | 25063.23 | 55 | 1149.16 | 0.00 |
| 10 | 25102.12 | 55 | 1188.05 | 0.00 |
| 0.5 | 25158.35 | 57 | 1244.28 | 0.00 |
| 8 | 25168.68 | 55 | 1254.61 | 0.00 |
| 0.2 | 25907.11 | 57 | 1993.04 | 0.00 |

**Supplementary Table S7.** The performance of general linear models describing the relative abundance of forest generalist birds in Pennsylvania, USA and containing landscape variables measured at different spatial scales. All models included the explanatory variables forest amount, forest patch density, forest clumpiness index, urbanization, agriculture amount, landscape heterogeneity 1, land use change, dominant habitat type, observer, start time, date, and year (see Table 1 for variable descriptions). Models at the 0.2 and 0.5 km scales also included the variable high intensity urbanization and models at the 0.2, 0.5, 1, 2, 4, and 6 km scales also included the variable landscape heterogeneity 2. K = the number of estimated parameters; AIC = Akaike's Information Criterion; $\Delta_{i}$ = AIC*_i_* – minAIC for each model *i*; w*_i_* = Akaike weight, or probability of being the best model given the observed data and the set of models evaluated.

| Scale (km) | AIC | K | $\Delta_{i}$ | w*_i_* |
| --- | --- | --- | --- | --- |
| 6 | 35983.81 | 56 | 0.00 | 1.00 |
| 4 | 36036.85 | 56 | 53.04 | 0.00 |
| 8 | 36072.81 | 55 | 89.00 | 0.00 |
| 16 | 36073.62 | 55 | 89.81 | 0.00 |
| 10 | 36074.89 | 55 | 91.08 | 0.00 |
| 12 | 36075.19 | 55 | 91.38 | 0.00 |
| 0.5 | 36085.52 | 57 | 101.71 | 0.00 |
| 2 | 36096.84 | 56 | 113.03 | 0.00 |
| 1 | 36099.02 | 56 | 115.21 | 0.00 |
| 0.2 | 36135.86 | 57 | 152.05 | 0.00 |

**Supplementary Table S8.** The performance of general linear models describing the species richness of forest generalist birds in Pennsylvania, USA and containing landscape variables measured at different spatial scales. All models included the explanatory variables forest amount, forest patch density, forest clumpiness index, urbanization, agriculture amount, landscape heterogeneity 1, land use change, dominant habitat type, observer, start time, date, and year (see Table 1 for variable descriptions). Models at the 0.2 and 0.5 km scales also included the variable high intensity urbanization and models at the 0.2, 0.5, 1, 2, 4, and 6 km scales also included the variable landscape heterogeneity 2. K = the number of estimated parameters; AIC = Akaike's Information Criterion; $\Delta_{i}$ = AIC*_i_* – minAIC for each model *i*; w*_i_* = Akaike weight, or probability of being the best model given the observed data and the set of models evaluated.

| Scale (km) | AIC | K | $\Delta_{i}$ | w*_i_* |
| --- | --- | --- | --- | --- |
| 10 | 63512.12 | 55 | 0.00 | 0.34 |
| 0.5 | 63513.16 | 57 | 1.04 | 0.20 |
| 8 | 63513.80 | 55 | 1.68 | 0.15 |
| 12 | 63514.04 | 55 | 1.92 | 0.13 |
| 16 | 63515.69 | 55 | 3.57 | 0.06 |
| 1 | 63515.91 | 56 | 3.79 | 0.05 |
| 4 | 63517.99 | 56 | 5.87 | 0.02 |
| 6 | 63518.16 | 56 | 6.04 | 0.02 |
| 2 | 63518.34 | 56 | 6.22 | 0.02 |
| 0.2 | 63518.78 | 57 | 6.66 | 0.01 |

**Supplementary Table S9.** The performance of general linear models describing the species evenness of forest generalist birds in Pennsylvania, USA and containing landscape variables measured at different spatial scales. All models included the explanatory variables forest amount, forest patch density, forest clumpiness index, urbanization, agriculture amount, landscape heterogeneity 1, land use change, dominant habitat type, observer, start time, date, and year (see Table 1 for variable descriptions). Models at the 0.2 and 0.5 km scales also included the variable high intensity urbanization and models at the 0.2, 0.5, 1, 2, 4, and 6 km scales also included the variable landscape heterogeneity 2. K = the number of estimated parameters; AIC = Akaike's Information Criterion; $\Delta_{i}$ = AIC*_i_* – minAIC for each model *i*; w*_i_* = Akaike weight, or probability of being the best model given the observed data and the set of models evaluated.

| Scale (km) | AIC | K | $\Delta_{i}$ | w*_i_* |
| --- | --- | --- | --- | --- |
| 0.2 | 38243.74 | 57 | 0.00 | 1.00 |
| 0.5 | 38269.93 | 57 | 26.19 | 0.00 |
| 1 | 38276.89 | 56 | 33.15 | 0.00 |
| 2 | 38289.02 | 56 | 45.28 | 0.00 |
| 4 | 38296.70 | 56 | 52.96 | 0.00 |
| 6 | 38301.92 | 56 | 58.18 | 0.00 |
| 8 | 38304.42 | 55 | 60.68 | 0.00 |
| 16 | 38304.90 | 55 | 61.16 | 0.00 |
| 10 | 38305.52 | 55 | 61.78 | 0.00 |
| 12 | 38305.89 | 55 | 62.15 | 0.00 |

**Supplementary Table S10.** Kolmogorov-Smirnov test statistic values for each of 101 bird species and 10 landscapes scales. Dark gray shading: *p* < 0.01; light gray shading: *p* < 0.05; no shading: *p* ≥ 0.05. Significant (*p* < 0.05) positive values indicate a large proportion of the individuals of a species occurred in the landscapes with the most forest, significant negative values indicate a large proportion of the individuals of a species occurred in the landscapes with the least forest, and non-significant values indicate a species occurred in proportion to forest cover. Species were classified into forest-area sensitive and edge/open country habitat association guilds based on whether the majority of their test statistic values across scales were significantly positive or significantly negative, respectively. Species that did not meet either of these criteria were classified as forest generalists.

| Guild | Species | Landscape scale (km) | | | | | | | | | |
| --- | --- | --- | --- | --- | --- | --- | --- | --- | --- | --- | --- |
|  |  | 0.2 | 0.5 | 1 | 2 | 4 | 6 | 8 | 10 | 12 | 16 |
| Forest-area sensitive | Black-throated Blue Warbler | 0.35 | 0.40 | 0.43 | 0.47 | 0.49 | 0.51 | 0.52 | 0.51 | 0.49 | 0.49 |
|  | (*Setophaga caerulescens*) |  |  |  |  |  |  |  |  |  |  |
| Forest-area sensitive | Canada Warbler | 0.32 | 0.41 | 0.39 | 0.40 | 0.43 | 0.45 | 0.46 | 0.42 | 0.40 | 0.37 |
|  | (*Cardellina canadensis*) |  |  |  |  |  |  |  |  |  |  |
| Forest-area sensitive | Winter Wren | 0.20 | 0.20 | 0.28 | 0.36 | 0.41 | 0.43 | 0.43 | 0.44 | 0.44 | 0.47 |
|  | (*Troglodytes hiemalis*) |  |  |  |  |  |  |  |  |  |  |
| Forest-area sensitive | Brown Creeper | 0.22 | 0.24 | 0.29 | 0.33 | 0.36 | 0.38 | 0.38 | 0.38 | 0.37 | 0.37 |
|  | (*Certhia americana*) |  |  |  |  |  |  |  |  |  |  |
| Forest-area sensitive | Blackburnian Warbler | 0.18 | 0.22 | 0.29 | 0.35 | 0.39 | 0.41 | 0.43 | 0.44 | 0.44 | 0.43 |
|  | (*Setophaga fusca*) |  |  |  |  |  |  |  |  |  |  |
| Forest-area sensitive | Magnolia Warbler | 0.18 | 0.21 | 0.25 | 0.31 | 0.36 | 0.38 | 0.39 | 0.40 | 0.40 | 0.41 |
|  | (*Setophaga magnolia*) |  |  |  |  |  |  |  |  |  |  |
| Forest-area sensitive | Golden-crowned Kinglet | 0.11 | 0.20 | 0.24 | 0.26 | 0.31 | 0.36 | 0.38 | 0.39 | 0.41 | 0.43 |
|  | (*Regulus satrapa*) |  |  |  |  |  |  |  |  |  |  |
| Forest-area sensitive | Blue-headed Vireo | 0.15 | 0.19 | 0.25 | 0.29 | 0.33 | 0.35 | 0.36 | 0.37 | 0.38 | 0.37 |
|  | (*Vireo solitarius*) |  |  |  |  |  |  |  |  |  |  |
| Forest-area sensitive | Hermit Thrush | 0.19 | 0.17 | 0.21 | 0.26 | 0.31 | 0.34 | 0.35 | 0.35 | 0.36 | 0.37 |
|  | (*Catharus guttatus*) |  |  |  |  |  |  |  |  |  |  |
| Forest-area sensitive | Dark-eyed Junco | 0.18 | 0.18 | 0.24 | 0.28 | 0.31 | 0.33 | 0.34 | 0.35 | 0.34 | 0.35 |
|  | (*Junco hyemalis*) |  |  |  |  |  |  |  |  |  |  |
| Forest-area sensitive | Black-throated Green Warbler | 0.14 | 0.19 | 0.21 | 0.25 | 0.28 | 0.31 | 0.31 | 0.32 | 0.32 | 0.32 |
|  | (*Setophaga virens*) |  |  |  |  |  |  |  |  |  |  |
| Forest-area sensitive | Red-breasted Nuthatch | 0.08 | 0.11 | 0.17 | 0.22 | 0.30 | 0.29 | 0.33 | 0.33 | 0.34 | 0.35 |
|  | (*Sitta canadensis*) |  |  |  |  |  |  |  |  |  |  |
| Forest-area sensitive | Black-and-white Warbler | -0.08 | 0.09 | 0.15 | 0.21 | 0.24 | 0.25 | 0.27 | 0.26 | 0.27 | 0.25 |
|  | (*Mniotilta varia*) |  |  |  |  |  |  |  |  |  |  |
| Forest-area sensitive | Common Raven | -0.13 | 0.11 | 0.13 | 0.19 | 0.22 | 0.24 | 0.25 | 0.26 | 0.27 | 0.29 |
|  | (*Corvus corax*) |  |  |  |  |  |  |  |  |  |  |
| Forest-area sensitive | Yellow-rumped Warbler | 0.07 | 0.09 | 0.16 | 0.19 | 0.25 | 0.27 | 0.28 | 0.29 | 0.29 | 0.31 |
|  | (*Setophaga coronata*) |  |  |  |  |  |  |  |  |  |  |
| Forest-area sensitive | Yellow-bellied Sapsucker | 0.15 | 0.11 | 0.14 | 0.18 | 0.22 | 0.25 | 0.27 | 0.28 | 0.29 | 0.31 |
|  | (*Sphyrapicus varius*) |  |  |  |  |  |  |  |  |  |  |
| Forest generalist | Golden-winged Warbler | -0.25 | -0.12 | -0.09 | 0.13 | 0.19 | 0.21 | 0.24 | 0.27 | 0.31 | 0.31 |
|  | (*Vermivora chrysoptera*) |  |  |  |  |  |  |  |  |  |  |
| Forest generalist | Least Flycatcher | -0.14 | 0.10 | 0.12 | 0.15 | 0.19 | 0.21 | 0.21 | 0.21 | 0.22 | 0.22 |
|  | (*Empidonax minimus*) |  |  |  |  |  |  |  |  |  |  |
| Forest generalist | Mourning Warbler | -0.19 | -0.07 | 0.10 | 0.14 | 0.17 | 0.20 | 0.22 | 0.23 | 0.25 | 0.26 |
|  | (*Geothlypis philadelphia*) |  |  |  |  |  |  |  |  |  |  |
| Forest generalist | Pine Warbler | -0.13 | -0.07 | 0.09 | 0.12 | 0.19 | 0.21 | 0.22 | 0.20 | 0.20 | 0.22 |
|  | (*Setophaga pinus*) |  |  |  |  |  |  |  |  |  |  |
| Forest generalist | Northern Parula | -0.22 | -0.12 | -0.06 | 0.08 | 0.13 | 0.15 | 0.17 | 0.17 | 0.16 | 0.16 |
|  | (*Setophaga americana*) |  |  |  |  |  |  |  |  |  |  |
| Forest generalist | Veery | -0.12 | 0.05 | 0.08 | 0.10 | 0.13 | 0.15 | 0.15 | 0.15 | 0.15 | 0.15 |
|  | (*Catharus fuscescens*) |  |  |  |  |  |  |  |  |  |  |
| Forest generalist | Cerulean Warbler | -0.13 | -0.06 | 0.07 | 0.11 | 0.16 | 0.16 | 0.17 | 0.15 | 0.15 | 0.13 |
|  | (*Setophaga cerulea*) |  |  |  |  |  |  |  |  |  |  |
| Forest generalist | Chestnut-sided Warbler | -0.12 | -0.03 | 0.05 | 0.08 | 0.11 | 0.12 | 0.13 | 0.14 | 0.15 | 0.16 |
|  | (*Setophaga pensylvanica*) |  |  |  |  |  |  |  |  |  |  |
| Forest generalist | Ovenbird | -0.13 | -0.06 | 0.02 | 0.05 | 0.08 | 0.09 | 0.10 | 0.10 | 0.10 | 0.10 |
|  | (*Seiurus aurocapillus*) |  |  |  |  |  |  |  |  |  |  |
| Forest generalist | American Redstart | -0.15 | -0.08 | -0.03 | 0.04 | 0.06 | 0.07 | 0.08 | 0.09 | 0.08 | 0.08 |
|  | (*Setophaga ruticilla*) |  |  |  |  |  |  |  |  |  |  |
| Forest generalist | Red-eyed Vireo | -0.17 | -0.08 | -0.04 | -0.01 | 0.04 | 0.05 | 0.06 | 0.06 | 0.07 | 0.07 |
|  | (*Vireo olivaceus*) |  |  |  |  |  |  |  |  |  |  |
| Forest generalist | Scarlet Tanager | -0.16 | -0.08 | -0.04 | -0.01 | 0.04 | 0.05 | 0.05 | 0.05 | 0.05 | 0.05 |
|  | (*Piranga olivacea*) |  |  |  |  |  |  |  |  |  |  |
| Forest generalist | Hooded Warbler | -0.15 | -0.08 | -0.04 | -0.02 | 0.03 | 0.04 | 0.04 | 0.04 | 0.04 | 0.04 |
|  | (*Setophaga citrina*) |  |  |  |  |  |  |  |  |  |  |
| Forest generalist | Louisiana Waterthrush | -0.19 | -0.10 | -0.06 | -0.03 | 0.07 | 0.07 | 0.08 | 0.07 | -0.07 | -0.08 |
|  | (*Parkesia motacilla*) |  |  |  |  |  |  |  |  |  |  |
| Forest generalist | Acadian Flycatcher | -0.16 | -0.08 | -0.04 | -0.03 | -0.02 | -0.03 | -0.04 | -0.05 | -0.05 | -0.07 |
|  | (*Empidonax virescens*) |  |  |  |  |  |  |  |  |  |  |
| Forest generalist | Pileated Woodpecker | -0.19 | -0.11 | -0.07 | -0.05 | -0.04 | -0.04 | -0.03 | -0.03 | -0.04 | -0.05 |
|  | (*Dryocopus pileatus*) |  |  |  |  |  |  |  |  |  |  |
| Forest generalist | Black-billed Cuckoo | -0.23 | -0.15 | -0.10 | -0.06 | -0.06 | 0.06 | 0.08 | 0.08 | 0.08 | 0.08 |
|  | (*Coccyzus erythropthalmus*) |  |  |  |  |  |  |  |  |  |  |
| Forest generalist | Black-capped Chickadee | -0.21 | -0.12 | -0.07 | -0.05 | -0.02 | 0.03 | 0.04 | 0.04 | 0.05 | 0.05 |
|  | (*Poecile atricapillus*) |  |  |  |  |  |  |  |  |  |  |
| Forest generalist | Ruby-throated Hummingbird | -0.22 | -0.14 | -0.09 | -0.06 | -0.03 | -0.03 | 0.01 | -0.01 | -0.01 | -0.01 |
|  | (*Archilochus colubris*) |  |  |  |  |  |  |  |  |  |  |
| Forest generalist | Hairy Woodpecker | -0.23 | -0.15 | -0.12 | -0.09 | -0.05 | -0.04 | -0.02 | -0.02 | -0.02 | 0.02 |
|  | (*Picoides villosus*) |  |  |  |  |  |  |  |  |  |  |
| Forest generalist | Rose-breasted Grosbeak | -0.22 | -0.14 | -0.09 | -0.08 | -0.06 | -0.05 | -0.05 | -0.05 | -0.04 | 0.04 |
|  | (*Pheucticus ludovicianus*) |  |  |  |  |  |  |  |  |  |  |
| Forest generalist | Eastern Towhee | -0.21 | -0.13 | -0.09 | -0.08 | -0.06 | -0.06 | -0.06 | -0.05 | -0.06 | -0.06 |
|  | (*Pipilo erythrophthalmus*) |  |  |  |  |  |  |  |  |  |  |
| Forest generalist | White-breasted Nuthatch | -0.23 | -0.15 | -0.11 | -0.08 | -0.07 | -0.06 | -0.06 | -0.04 | -0.04 | -0.04 |
|  | (*Sitta carolinensis*) |  |  |  |  |  |  |  |  |  |  |
| Forest generalist | Yellow-billed Cuckoo | -0.24 | -0.16 | -0.11 | -0.08 | -0.06 | -0.05 | -0.05 | -0.05 | -0.05 | -0.05 |
|  | (*Coccyzus americanus*) |  |  |  |  |  |  |  |  |  |  |
| Forest generalist | Worm-eating Warbler | -0.24 | -0.16 | -0.12 | -0.07 | -0.05 | -0.06 | -0.07 | -0.08 | -0.08 | -0.08 |
|  | (*Helmitheros vermivorum*) |  |  |  |  |  |  |  |  |  |  |
| Forest generalist | Blue-gray Gnatcatcher | -0.24 | -0.18 | -0.14 | -0.10 | -0.09 | -0.08 | -0.08 | -0.08 | -0.08 | -0.08 |
|  | (*Polioptila caerulea*) |  |  |  |  |  |  |  |  |  |  |
| Forest generalist | Prairie Warbler | -0.26 | -0.18 | -0.15 | -0.13 | -0.15 | -0.15 | -0.14 | -0.15 | -0.14 | -0.14 |
|  | (*Setophaga discolor*) |  |  |  |  |  |  |  |  |  |  |
| Forest generalist | Purple Finch | -0.32 | -0.19 | -0.12 | -0.08 | -0.04 | -0.05 | -0.04 | 0.04 | 0.06 | 0.07 |
|  | (*Haemorhous purpureus*) |  |  |  |  |  |  |  |  |  |  |
| Forest generalist | Common Yellowthroat | -0.27 | -0.19 | -0.16 | -0.14 | -0.12 | -0.11 | -0.10 | -0.09 | -0.09 | -0.08 |
|  | (*Geothlypis trichas*) |  |  |  |  |  |  |  |  |  |  |
| Forest generalist | Indigo Bunting | -0.30 | -0.22 | -0.18 | -0.16 | -0.13 | -0.11 | -0.10 | -0.10 | -0.09 | -0.08 |
|  | (*Passerina cyanea*) |  |  |  |  |  |  |  |  |  |  |
| Forest generalist | Eastern Phoebe | -0.29 | -0.20 | -0.17 | -0.15 | -0.13 | -0.13 | -0.12 | -0.11 | -0.11 | -0.09 |
|  | (*Sayornis phoebe*) |  |  |  |  |  |  |  |  |  |  |
| Forest generalist | Cedar Waxwing | -0.30 | -0.23 | -0.19 | -0.16 | -0.13 | -0.13 | -0.12 | -0.11 | -0.11 | -0.09 |
|  | (*Bombycilla cedrorum*) |  |  |  |  |  |  |  |  |  |  |
| Forest generalist | Kentucky Warbler | -0.26 | -0.19 | -0.16 | -0.17 | -0.15 | -0.15 | -0.13 | -0.13 | -0.14 | -0.13 |
|  | (*Geothlypis formosa*) |  |  |  |  |  |  |  |  |  |  |
| Forest generalist | Eastern Wood-Pewee | -0.28 | -0.21 | -0.19 | -0.16 | -0.15 | -0.14 | -0.15 | -0.15 | -0.15 | -0.17 |
|  | (*Contopus virens*) |  |  |  |  |  |  |  |  |  |  |
| Forest generalist | American Crow | -0.31 | -0.24 | -0.20 | -0.18 | -0.16 | -0.15 | -0.15 | -0.14 | -0.13 | -0.12 |
|  | (*Corvus brachyrhynchos*) |  |  |  |  |  |  |  |  |  |  |
| Forest generalist | Yellow-throated Warbler | -0.26 | -0.23 | -0.20 | -0.17 | -0.19 | -0.16 | -0.15 | -0.14 | -0.16 | -0.15 |
|  | (*Setophaga dominica*) |  |  |  |  |  |  |  |  |  |  |
| Forest generalist | Chipping Sparrow | -0.33 | -0.25 | -0.22 | -0.19 | -0.17 | -0.16 | -0.15 | -0.14 | -0.13 | -0.13 |
|  | (*Spizella passerina*) |  |  |  |  |  |  |  |  |  |  |
| Forest generalist | Tufted Titmouse | -0.30 | -0.24 | -0.21 | -0.19 | -0.17 | -0.17 | -0.17 | -0.16 | -0.16 | -0.16 |
|  | (*Baeolophus bicolor*) |  |  |  |  |  |  |  |  |  |  |
| Forest generalist | Wood Thrush | -0.29 | -0.23 | -0.20 | -0.19 | -0.18 | -0.18 | -0.18 | -0.18 | -0.18 | -0.19 |
|  | (*Hylocichla mustelina*) |  |  |  |  |  |  |  |  |  |  |
| Forest generalist | Swamp Sparrow | -0.36 | -0.29 | -0.22 | -0.19 | -0.16 | -0.15 | -0.15 | -0.15 | -0.17 | -0.17 |
|  | (*Melospiza georgiana*) |  |  |  |  |  |  |  |  |  |  |
| Forest generalist | Northern Flicker | -0.32 | -0.24 | -0.21 | -0.20 | -0.17 | -0.16 | -0.16 | -0.15 | -0.15 | -0.14 |
|  | (*Colaptes auratus*) |  |  |  |  |  |  |  |  |  |  |
| Forest generalist | Great Crested Flycatcher | -0.34 | -0.27 | -0.24 | -0.21 | -0.18 | -0.16 | -0.16 | -0.15 | -0.14 | -0.14 |
|  | (*Myiarchus crinitus*) |  |  |  |  |  |  |  |  |  |  |
| Forest generalist | Tree Swallow | -0.37 | -0.30 | -0.26 | -0.22 | -0.19 | -0.18 | -0.17 | -0.16 | -0.15 | -0.14 |
|  | (*Tachycineta bicolor*) |  |  |  |  |  |  |  |  |  |  |
| Edge/open country | Field Sparrow | -0.34 | -0.28 | -0.23 | -0.21 | -0.20 | -0.19 | -0.17 | -0.16 | -0.15 | -0.15 |
|  | (*Spizella pusilla*) |  |  |  |  |  |  |  |  |  |  |
| Edge/open country | Northern Rough-winged Swallow | -0.41 | -0.32 | -0.29 | -0.22 | -0.20 | -0.16 | -0.18 | -0.17 | -0.18 | -0.16 |
|  | (*Stelgidopteryx serripennis*) |  |  |  |  |  |  |  |  |  |  |
| Edge/open country | Yellow-breasted Chat | -0.30 | -0.24 | -0.22 | -0.22 | -0.18 | -0.18 | -0.21 | -0.20 | -0.19 | -0.15 |
|  | (*Icteria virens*) |  |  |  |  |  |  |  |  |  |  |
| Edge/open country | Blue Jay | -0.33 | -0.27 | -0.24 | -0.22 | -0.21 | -0.20 | -0.19 | -0.19 | -0.18 | -0.18 |
|  | (*Cyanocitta cristata*) |  |  |  |  |  |  |  |  |  |  |
| Edge/open country | Brown Thrasher | -0.37 | -0.30 | -0.27 | -0.26 | -0.23 | -0.23 | -0.21 | -0.20 | -0.19 | -0.17 |
|  | (*Toxostoma rufum*) |  |  |  |  |  |  |  |  |  |  |
| Edge/open country | Henslow's Sparrow | -0.40 | -0.35 | -0.31 | -0.23 | -0.19 | -0.20 | -0.20 | -0.22 | -0.19 | -0.25 |
|  | (*Ammodramus henslowii*) |  |  |  |  |  |  |  |  |  |  |
| Edge/open country | Grasshopper Sparrow | -0.44 | -0.35 | -0.31 | -0.26 | -0.23 | -0.22 | -0.21 | -0.19 | -0.19 | -0.19 |
|  | (*Ammodramus savannarum*) |  |  |  |  |  |  |  |  |  |  |
| Edge/open country | Alder Flycatcher | -0.34 | -0.27 | -0.24 | -0.24 | -0.24 | -0.23 | -0.23 | -0.20 | -0.20 | -0.19 |
|  | (*Empidonax alnorum*) |  |  |  |  |  |  |  |  |  |  |
| Edge/open country | Blue-winged Warbler | -0.29 | -0.21 | -0.20 | -0.21 | -0.20 | -0.22 | -0.22 | -0.23 | -0.23 | -0.24 |
|  | (*Vermivora cyanoptera*) |  |  |  |  |  |  |  |  |  |  |
| Edge/open country | Downy Woodpecker | -0.34 | -0.28 | -0.26 | -0.24 | -0.22 | -0.21 | -0.21 | -0.21 | -0.21 | -0.21 |
|  | (*Picoides pubescens*) |  |  |  |  |  |  |  |  |  |  |
| Edge/open country | Baltimore Oriole | -0.37 | -0.31 | -0.28 | -0.25 | -0.24 | -0.24 | -0.24 | -0.24 | -0.23 | -0.22 |
|  | (*Icterus galbula*) |  |  |  |  |  |  |  |  |  |  |
| Edge/open country | American Goldfinch | -0.38 | -0.31 | -0.28 | -0.27 | -0.25 | -0.24 | -0.23 | -0.23 | -0.22 | -0.21 |
|  | (*Spinus tristis*) |  |  |  |  |  |  |  |  |  |  |
| Edge/open country | Eastern Bluebird | -0.41 | -0.34 | -0.30 | -0.28 | -0.25 | -0.25 | -0.24 | -0.23 | -0.22 | -0.20 |
|  | (*Sialia sialis*) |  |  |  |  |  |  |  |  |  |  |
| Edge/open country | Gray Catbird | -0.38 | -0.32 | -0.29 | -0.28 | -0.26 | -0.26 | -0.26 | -0.25 | -0.25 | -0.25 |
|  | (*Dumetella carolinensis*) |  |  |  |  |  |  |  |  |  |  |
| Edge/open country | Song Sparrow | -0.40 | -0.33 | -0.30 | -0.29 | -0.27 | -0.26 | -0.25 | -0.25 | -0.24 | -0.22 |
|  | (*Melospiza melodia*) |  |  |  |  |  |  |  |  |  |  |
| Edge/open country | Yellow Warbler | -0.39 | -0.33 | -0.29 | -0.29 | -0.28 | -0.27 | -0.27 | -0.26 | -0.25 | -0.24 |
|  | (*Setophaga petechia*) |  |  |  |  |  |  |  |  |  |  |
| Edge/open country | Orchard Oriole | -0.33 | -0.30 | -0.30 | -0.28 | -0.27 | -0.28 | -0.29 | -0.28 | -0.27 | -0.26 |
|  | (*Icterus spurius*) |  |  |  |  |  |  |  |  |  |  |
| Edge/open country | Bobolink | -0.44 | -0.36 | -0.31 | -0.30 | -0.28 | -0.26 | -0.26 | -0.24 | -0.22 | -0.21 |
|  | (*Dolichonyx oryzivorus*) |  |  |  |  |  |  |  |  |  |  |
| Edge/open country | Mourning Dove | -0.40 | -0.34 | -0.31 | -0.29 | -0.27 | -0.27 | -0.26 | -0.25 | -0.25 | -0.24 |
|  | (*Zenaida macroura*) |  |  |  |  |  |  |  |  |  |  |
| Edge/open country | Barn Swallow | -0.43 | -0.36 | -0.32 | -0.30 | -0.28 | -0.27 | -0.25 | -0.25 | -0.23 | -0.21 |
|  | (*Hirundo rustica*) |  |  |  |  |  |  |  |  |  |  |
| Edge/open country | American Robin | -0.40 | -0.34 | -0.31 | -0.30 | -0.28 | -0.27 | -0.27 | -0.26 | -0.25 | -0.24 |
|  | (*Turdus migratorius*) |  |  |  |  |  |  |  |  |  |  |
| Edge/open country | Eastern Meadowlark | -0.45 | -0.37 | -0.33 | -0.31 | -0.29 | -0.28 | -0.27 | -0.25 | -0.24 | -0.22 |
|  | (*Sturnella magna*) |  |  |  |  |  |  |  |  |  |  |
| Edge/open country | Brown-headed Cowbird | -0.40 | -0.34 | -0.32 | -0.30 | -0.29 | -0.29 | -0.29 | -0.28 | -0.28 | -0.28 |
|  | (*Molothrus ater*) |  |  |  |  |  |  |  |  |  |  |
| Edge/open country | White-eyed Vireo | -0.37 | -0.29 | -0.28 | -0.28 | -0.30 | -0.31 | -0.30 | -0.30 | -0.32 | -0.36 |
|  | (*Vireo griseus*) |  |  |  |  |  |  |  |  |  |  |
| Edge/open country | Red-winged Blackbird | -0.44 | -0.37 | -0.34 | -0.32 | -0.31 | -0.30 | -0.29 | -0.28 | -0.27 | -0.25 |
|  | (*Agelaius phoeniceus*) |  |  |  |  |  |  |  |  |  |  |
| Edge/open country | Northern Cardinal | -0.41 | -0.35 | -0.33 | -0.32 | -0.32 | -0.32 | -0.31 | -0.31 | -0.31 | -0.31 |
|  | (*Cardinalis cardinalis*) |  |  |  |  |  |  |  |  |  |  |
| Edge/open country | House Wren | -0.43 | -0.38 | -0.35 | -0.34 | -0.33 | -0.32 | -0.32 | -0.31 | -0.30 | -0.30 |
|  | (*Troglodytes aedon*) |  |  |  |  |  |  |  |  |  |  |
| Edge/open country | Red-bellied Woodpecker | -0.42 | -0.37 | -0.34 | -0.33 | -0.32 | -0.32 | -0.33 | -0.33 | -0.33 | -0.34 |
|  | (*Melanerpes carolinus*) |  |  |  |  |  |  |  |  |  |  |
| Edge/open country | Killdeer | -0.46 | -0.40 | -0.38 | -0.36 | -0.34 | -0.33 | -0.32 | -0.31 | -0.30 | -0.28 |
|  | (*Charadrius vociferus*) |  |  |  |  |  |  |  |  |  |  |
| Edge/open country | Common Grackle | -0.45 | -0.40 | -0.37 | -0.36 | -0.34 | -0.33 | -0.32 | -0.31 | -0.30 | -0.29 |
|  | (*Quiscalus quiscula*) |  |  |  |  |  |  |  |  |  |  |
| Edge/open country | Chimney Swift | -0.43 | -0.38 | -0.36 | -0.36 | -0.36 | -0.35 | -0.34 | -0.34 | -0.33 | -0.32 |
|  | (*Chaetura pelagica*) |  |  |  |  |  |  |  |  |  |  |
| Edge/open country | Eastern Kingbird | -0.47 | -0.40 | -0.37 | -0.36 | -0.34 | -0.33 | -0.33 | -0.33 | -0.33 | -0.32 |
|  | (*Tyrannus tyrannus*) |  |  |  |  |  |  |  |  |  |  |
| Edge/open country | European Starling | -0.47 | -0.42 | -0.39 | -0.37 | -0.34 | -0.34 | -0.33 | -0.32 | -0.31 | -0.30 |
|  | (*Sturnus vulgaris*) |  |  |  |  |  |  |  |  |  |  |
| Edge/open country | Savannah Sparrow | -0.50 | -0.43 | -0.41 | -0.37 | -0.36 | -0.35 | -0.34 | -0.33 | -0.31 | -0.27 |
|  | (*Passerculus sandwichensis*) |  |  |  |  |  |  |  |  |  |  |
| Edge/open country | Carolina Wren | -0.42 | -0.38 | -0.37 | -0.37 | -0.37 | -0.37 | -0.37 | -0.37 | -0.37 | -0.38 |
|  | (*Thryothorus ludovicianus*) |  |  |  |  |  |  |  |  |  |  |
| Edge/open country | Warbling Vireo | -0.47 | -0.41 | -0.38 | -0.38 | -0.38 | -0.37 | -0.36 | -0.36 | -0.35 | -0.35 |
|  | (*Vireo gilvus*) |  |  |  |  |  |  |  |  |  |  |
| Edge/open country | Rock Pigeon | -0.55 | -0.47 | -0.45 | -0.42 | -0.38 | -0.37 | -0.35 | -0.33 | -0.30 | -0.29 |
|  | (*Columba livia*) |  |  |  |  |  |  |  |  |  |  |
| Edge/open country | Willow Flycatcher | -0.46 | -0.40 | -0.38 | -0.39 | -0.40 | -0.39 | -0.38 | -0.37 | -0.36 | -0.35 |
|  | (*Empidonax traillii*) |  |  |  |  |  |  |  |  |  |  |
| Edge/open country | House Sparrow | -0.50 | -0.45 | -0.44 | -0.42 | -0.41 | -0.40 | -0.39 | -0.38 | -0.37 | -0.36 |
|  | (*Passer domesticus*) |  |  |  |  |  |  |  |  |  |  |
| Edge/open country | House Finch | -0.50 | -0.46 | -0.44 | -0.43 | -0.42 | -0.41 | -0.40 | -0.40 | -0.40 | -0.39 |
|  | (*Haemorhous mexicanus*) |  |  |  |  |  |  |  |  |  |  |
| Edge/open country | Northern Mockingbird | -0.51 | -0.49 | -0.48 | -0.48 | -0.47 | -0.47 | -0.46 | -0.47 | -0.47 | -0.46 |
|  | (*Mimus polyglottos*) |  |  |  |  |  |  |  |  |  |  |
| Edge/open country | Carolina Chickadee | -0.52 | -0.50 | -0.52 | -0.54 | -0.57 | -0.60 | -0.61 | -0.62 | -0.63 | -0.64 |
|  | (*Poecile carolinensis*) |  |  |  |  |  |  |  |  |  |  |

**Supplementary Table S11.** Pairwise correlations (Pearson's r) for all continuous explanatory variables. Landscape variables were measured at the 0.2 km scale.

|  | Forest amount | Forest patch density | Forest clumpiness index | Agriculture amount | Day | Start time | Urbanization | High intensity urbanization | Landscape heterogeneity 1 | Landscape heterogeneity 2 |
| --- | --- | --- | --- | --- | --- | --- | --- | --- | --- | --- |
| Forest amount | 1.00 |  |  |  |  |  |  |  |  |  |
| Forest patch density | -0.55 | 1.00 |  |  |  |  |  |  |  |  |
| Forest clumpiness index | 0.67 | -0.45 | 1.00 |  |  |  |  |  |  |  |
| Agriculture amount | -0.65 | 0.26 | -0.34 | 1.00 |  |  |  |  |  |  |
| Day | 0.11 | -0.07 | 0.15 | -0.08 | 1.00 |  |  |  |  |  |
| Start time | 0.01 | -0.02 | 0.00 | 0.00 | -0.02 | 1.00 |  |  |  |  |
| Urbanization | 0.38 | -0.23 | 0.23 | -0.01 | 0.13 | 0.01 | 1.00 |  |  |  |
| High intensity urbanization | -0.09 | 0.08 | -0.18 | 0.04 | -0.04 | 0.00 | -0.01 | 1.00 |  |  |
| Landscape heterogeneity 1 | 0.17 | -0.08 | 0.21 | -0.1 | 0.18 | 0.01 | 0.14 | -0.08 | 1.00 |  |
| Landscape heterogeneity 2 | 0.00 | 0.00 | -0.02 | 0.00 | -0.11 | 0.00 | -0.04 | 0.00 | 0.00 | 1.00 |

**Supplementary Table S12.** Pairwise correlations (Pearson's r) for all continuous explanatory variables. Landscape variables were measured at the 1 km scale.

|  | Forest amount | Forest patch density | Forest clumpiness index | Agriculture amount | Day | Start time | Urbanization | Landscape heterogeneity 1 | Landscape heterogeneity 2 |
| --- | --- | --- | --- | --- | --- | --- | --- | --- | --- |
| Forest amount | 1.00 |  |  |  |  |  |  |  |  |
| Forest patch density | -0.81 | 1.00 |  |  |  |  |  |  |  |
| Forest clumpiness index | 0.71 | -0.66 | 1.00 |  |  |  |  |  |  |
| Agriculture amount | -0.81 | 0.65 | -0.53 | 1.00 |  |  |  |  |  |
| Day | 0.14 | -0.13 | 0.15 | -0.14 | 1.00 |  |  |  |  |
| Start time | 0.01 | -0.01 | 0.01 | 0.00 | -0.02 | 1.00 |  |  |  |
| Urbanization | 0.46 | -0.37 | 0.33 | -0.05 | 0.14 | 0.01 | 1.00 |  |  |
| Landscape heterogeneity 1 | 0.74 | -0.61 | 0.74 | -0.51 | 0.15 | 0.02 | 0.46 | 1.00 |  |
| Landscape heterogeneity 2 | 0.23 | -0.13 | -0.07 | -0.28 | -0.24 | 0.01 | -0.19 | 0.03 | 1.00 |

**Supplementary Table S13.** Pairwise correlations (Pearson's r) for all continuous explanatory variables. Landscape variables were measured at the 6 km scale.

|  | Forest amount | Forest patch density | Forest clumpiness index | Agriculture amount | Day | Start time | Urbanization | Landscape heterogeneity 1 | Landscape heterogeneity 2 |
| --- | --- | --- | --- | --- | --- | --- | --- | --- | --- |
| Forest amount | 1.00 |  |  |  |  |  |  |  |  |
| Forest patch density | -0.92 | 1.00 |  |  |  |  |  |  |  |
| Forest clumpiness index | 0.84 | -0.84 | 1.00 |  |  |  |  |  |  |
| Agriculture amount | -0.83 | 0.75 | -0.66 | 1.00 |  |  |  |  |  |
| Day | 0.23 | -0.22 | 0.20 | -0.22 | 1.00 |  |  |  |  |
| Start time | 0.01 | -0.02 | 0.00 | 0.00 | -0.02 | 1.00 |  |  |  |
| Urbanization | 0.55 | -0.52 | 0.48 | -0.09 | 0.17 | 0.02 | 1.00 |  |  |
| Landscape heterogeneity 1 | 0.81 | -0.76 | 0.82 | -0.56 | 0.16 | 0.02 | 0.56 | 1.00 |  |
| Landscape heterogeneity 2 | -0.14 | 0.05 | 0.07 | 0.21 | 0.28 | -0.02 | 0.25 | 0.00 | 1.00 |

**Supplementary Table S14.** Pairwise correlations (Pearson's r) for all continuous explanatory variables. Landscape variables were measured at the 10 km scale.

|  | Forest amount | Forest patch density | Forest clumpiness index | Agriculture amount | Day | Start time | Urbanization | Landscape heterogeneity 1 |
| --- | --- | --- | --- | --- | --- | --- | --- | --- |
| Forest amount | 1.00 |  |  |  |  |  |  |  |
| Forest patch density | -0.93 | 1.00 |  |  |  |  |  |  |
| Forest clumpiness index | 0.83 | -0.84 | 1.00 |  |  |  |  |  |
| Agriculture amount | -0.84 | 0.75 | -0.63 | 1.00 |  |  |  |  |
| Day | 0.25 | -0.24 | 0.22 | -0.24 | 1.00 |  |  |  |
| Start time | 0.01 | -0.01 | -0.01 | 0.00 | -0.02 | 1.00 |  |  |
| Urbanization | 0.59 | -0.57 | 0.53 | -0.13 | 0.19 | 0.01 | 1.00 |  |
| Landscape heterogeneity 1 | 0.81 | -0.77 | 0.81 | -0.56 | 0.17 | 0.01 | 0.61 | 1.00 |

**Supplementary Table S15.** Variance inflation factors (VIF) of the explanatory variables in the best model of the relative abundance of all forest birds in Pennsylvania, USA. Landscape variables were measured at the 0.2 km scale.

| Variable | VIF |
| --- | --- |
| Observer | 6.10 |
| Day | 1.18 |
| Year | 3.83 |
| Start time | 1.03 |
| Land use change | 1.05 |
| Dominant habitat type | 2.04 |
| Landscape heterogeneity 1 | 1.18 |
| Landscape heterogeneity 2 | 1.06 |
| Forest amount | 4.14 |
| Agriculture amount | 2.19 |
| Forest patch density | 1.50 |
| Forest clumpiness index | 2.06 |
| Urbanization | 1.50 |
| High intensity urbanization | 1.13 |

**Supplementary Table S16.** Variance inflation factors (VIF) of the explanatory variables in the best model of the species richness of all forest birds in Pennsylvania, USA. Landscape variables were measured at the 0.2 km scale.

| Variable | VIF |
| --- | --- |
| Observer | 6.10 |
| Day | 1.18 |
| Year | 3.83 |
| Start time | 1.03 |
| Land use change | 1.05 |
| Dominant habitat type | 2.04 |
| Landscape heterogeneity 1 | 1.18 |
| Landscape heterogeneity 2 | 1.06 |
| Forest amount | 4.14 |
| Agriculture amount | 2.19 |
| Forest patch density | 1.50 |
| Forest clumpiness index | 2.06 |
| Urbanization | 1.50 |
| High intensity urbanization | 1.13 |

**Supplementary Table S17.** Variance inflation factors (VIF) of the explanatory variables in the best model of the species evenness of all forest birds in Pennsylvania, USA. Landscape variables were measured at the 0.2 km scale.

| Variable | VIF |
| --- | --- |
| Observer | 6.09 |
| Day | 1.18 |
| Year | 3.83 |
| Start time | 1.03 |
| Land use change | 1.05 |
| Dominant habitat type | 2.04 |
| Landscape heterogeneity 1 | 1.18 |
| Landscape heterogeneity 2 | 1.06 |
| Forest amount | 4.14 |
| Agriculture amount | 2.19 |
| Forest patch density | 1.50 |
| Forest clumpiness index | 2.06 |
| Urbanization | 1.50 |
| High intensity urbanization | 1.13 |

**Supplementary Table S18.** Variance inflation factors (VIF) of the explanatory variables in the best model of the relative abundance of forest-area sensitive birds in Pennsylvania, USA. Landscape variables were measured at the 6 km scale.

| Variable | VIF |
| --- | --- |
| Observer | 10.58 |
| Day | 1.39 |
| Year | 4.05 |
| Start time | 1.03 |
| Land use change | 1.06 |
| Dominant habitat type | 1.57 |
| Landscape heterogeneity 1 | 4.51 |
| Landscape heterogeneity 2 | 2.18 |
| Forest amount | 31.99 |
| Agriculture amount | 11.39 |
| Forest patch density | 7.95 |
| Forest clumpiness index | 6.86 |
| Urbanization | 5.39 |

**Supplementary Table S19.** Variance inflation factors (VIF) of the explanatory variables in the best model of the species richness of forest-area sensitive birds in Pennsylvania, USA. Landscape variables were measured at the 1 km scale.

| Variable | VIF |
| --- | --- |
| Observer | 7.07 |
| Day | 1.26 |
| Year | 3.95 |
| Start time | 1.03 |
| Land use change | 1.05 |
| Dominant habitat type | 1.67 |
| Landscape heterogeneity 1 | 3.26 |
| Landscape heterogeneity 2 | 1.72 |
| Forest amount | 11.32 |
| Agriculture amount | 5.14 |
| Forest patch density | 3.00 |
| Forest clumpiness index | 3.27 |
| Urbanization | 2.52 |

**Supplementary Table S20.** Variance inflation factors (VIF) of the explanatory variables in the best model of the species evenness of forest-area sensitive birds in Pennsylvania, USA. Landscape variables were measured at the 6 km scale.

| Variable | VIF |
| --- | --- |
| Observer | 11.24 |
| Day | 1.39 |
| Year | 4.24 |
| Start time | 1.03 |
| Land use change | 1.06 |
| Dominant habitat type | 1.63 |
| Landscape heterogeneity 1 | 4.75 |
| Landscape heterogeneity 2 | 2.10 |
| Forest amount | 32.97 |
| Agriculture amount | 11.77 |
| Forest patch density | 8.02 |
| Forest clumpiness index | 6.88 |
| Urbanization | 5.86 |

**Supplementary Table S21.** Variance inflation factors (VIF) of the explanatory variables in the best model of the relative abundance of forest generalist birds in Pennsylvania, USA. Landscape variables were measured at the 6 km scale.

| Variable | VIF |
| --- | --- |
| Observer | 10.58 |
| Day | 1.39 |
| Year | 4.05 |
| Start time | 1.03 |
| Land use change | 1.06 |
| Dominant habitat type | 1.57 |
| Landscape heterogeneity 1 | 4.51 |
| Landscape heterogeneity 2 | 2.18 |
| Forest amount | 31.99 |
| Agriculture amount | 11.39 |
| Forest patch density | 7.95 |
| Forest clumpiness index | 6.86 |
| Urbanization | 5.39 |

**Supplementary Table S22.** Variance inflation factors (VIF) of the explanatory variables in the best model of the species richness of forest generalist birds in Pennsylvania, USA. Landscape variables were measured at the 10 km scale.

| Variable | VIF |
| --- | --- |
| Observer | 10.61 |
| Day | 1.25 |
| Year | 4.06 |
| Start time | 1.03 |
| Land use change | 1.05 |
| Dominant habitat type | 1.52 |
| Landscape heterogeneity 1 | 4.58 |
| Forest amount | 30.78 |
| Agriculture amount | 12.77 |
| Forest patch density | 9.67 |
| Forest clumpiness index | 5.75 |
| Urbanization | 5.33 |

**Supplementary Table S23.** Variance inflation factors (VIF) of the explanatory variables in the best model of the species evenness of forest generalist birds in Pennsylvania, USA. Landscape variables were measured at the 0.2 km scale.

| Variable | VIF |
| --- | --- |
| Observer | 6.03 |
| Day | 1.18 |
| Year | 3.81 |
| Start time | 1.03 |
| Land use change | 1.05 |
| Dominant habitat type | 1.98 |
| Landscape heterogeneity 1 | 1.17 |
| Landscape heterogeneity 2 | 1.06 |
| Forest amount | 4.13 |
| Agriculture amount | 2.19 |
| Forest patch density | 1.49 |
| Forest clumpiness index | 2.05 |
| Urbanization | 1.49 |
| High intensity urbanization | 1.11 |
